# EHMT2 suppresses the variation of transcriptional switches in the mouse embryo

**DOI:** 10.1371/journal.pgen.1009908

**Published:** 2021-11-18

**Authors:** Tie-Bo Zeng, Nicholas Pierce, Ji Liao, Purnima Singh, Kin Lau, Wanding Zhou, Piroska E. Szabó

**Affiliations:** 1 Department of Epigenetics, Van Andel Institute, Grand Rapids, Michigan, United States of America; 2 Division of Molecular and Cellular Biology, City of Hope Cancer Center, Duarte, California, United States of America; 3 Bioinformatics and Biostatistics Core, Van Andel Institute, Grand Rapids, Michigan, United States of America; Walter and Eliza Hall Institute of Medical Research, AUSTRALIA

## Abstract

EHMT2 is the main euchromatic H3K9 methyltransferase. Embryos with zygotic, or maternal mutation in the *Ehmt2* gene exhibit variable developmental delay. To understand how EHMT2 prevents variable developmental delay we performed RNA sequencing of mutant and somite stage-matched normal embryos at 8.5–9.5 days of gestation. Using four-way comparisons between delayed and normal embryos we clarified what it takes to be normal and what it takes to develop. We identified differentially expressed genes, for example *Hox* genes that simply reflected the difference in developmental progression of wild type and the delayed mutant uterus-mate embryos. By comparing wild type and zygotic mutant embryos along the same developmental window we detected a role of EHMT2 in suppressing variation in the transcriptional switches. We identified transcription changes where precise switching during development occurred only in the normal but not in the mutant embryo. At the 6-somite stage, gastrulation-specific genes were not precisely switched off in the *Ehmt2*^*−/−*^ zygotic mutant embryos, while genes involved in organ growth, connective tissue development, striated muscle development, muscle differentiation, and cartilage development were not precisely switched on. The *Ehmt2*^*mat−/+*^ maternal mutant embryos displayed high transcriptional variation consistent with their variable survival. Variable derepression of transcripts occurred dominantly in the maternally inherited allele. Transcription was normal in the parental haploinsufficient wild type embryos despite their delay, consistent with their good prospects. Global profiling of transposable elements revealed EHMT2 targeted DNA methylation and suppression at LTR repeats, mostly ERVKs. In *Ehmt2*^*−/−*^ embryos, transcription over very long distances initiated from such misregulated ‘driver’ ERVK repeats, encompassing a multitude of misexpressed ‘passenger’ repeats. In summary, EHMT2 reduced transcriptional variation of developmental switch genes and developmentally switching repeat elements at the six-somite stage embryos. These findings establish EHMT2 as a suppressor of transcriptional and developmental variation at the transition between gastrulation and organ specification.

## Author summary

Developmental variation is the property of normal development, and its regulation is poorly understood. Variable developmental delay is found in embryos that carry mutations of epigenetic modifiers, suggesting a role of chromatin in controlling developmental delay and its variable nature. We analyzed a genetic series of mutations and found that EHMT2 suppresses variation of developmental delay and also suppresses the variation of transcriptional switches at the transition between gastrulation and organ specification.

## Introduction

**Fig 1:**
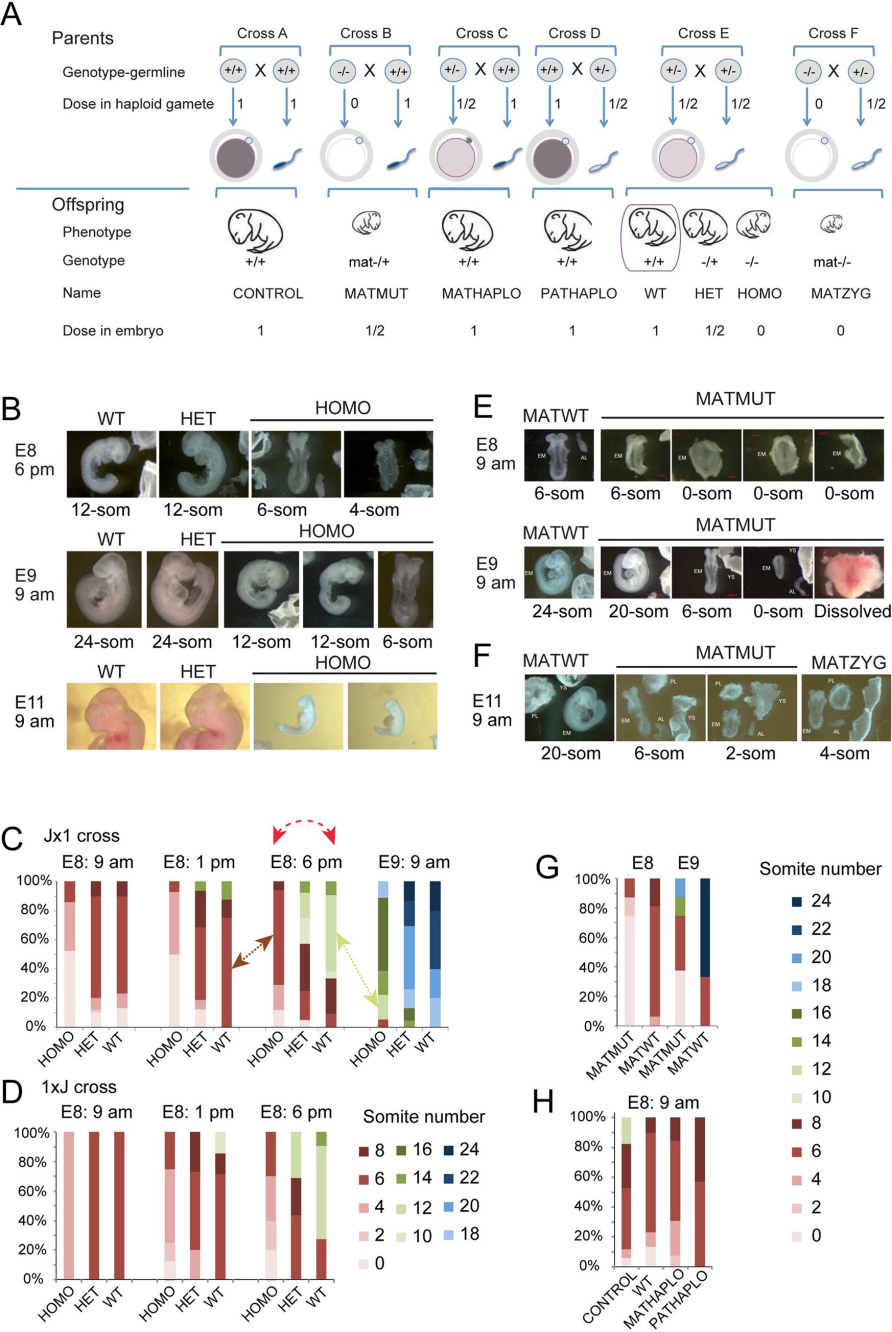
Zygotic and parental genetic deficiencies in *Ehmt2* result in delayed embryo development. (**A**) Study design. Genetic crosses to result in parental genotypes, and embryonic genotypes with insufficiencies of the epigenetic modifier in the gametes or in the embryo. We summarize the genetic crosses, the genotype of the parents (their diploid germ line), the dose of the epigenetic factor in the gametes and in the embryo, and the genotype of the embryo. We provide simple names for these embryo classes. Note, for example, that the WT embryo from the conventional test cross (purple box) is biparentally haploinsufficient. (**B-D**) Zygotic effect. (**B**) Images of representative embryos from crossing *Ehmt2*^+/−^ parents are shown, with the embryonic days and times of collection marked to the left. The embryo names are marked at the top of each image and the somite numbers are marked underneath each image. (**C**) Percentages of the embryos that have reached specific somite numbers at collection are tallied from the *Ehmt2*^+/−^ x *Ehmt2*^+/−^ cross in the JF1x129 parental strain background. Day and time of collection is indicated above the bars. Red dashed arrow shows that comparing uterus-mate embryos according to conventional methodology means comparing more advanced wild type and delayed mutant embryos, and each group has its variation. The brown and green arrows indicate comparing somite-matched embryos. The number of pregnant mothers and embryos were: E8: 9 am (n = 17, 91), E8: 1 pm (n = 7, 38), E8: 6 pm (n = 14, 78) and E9: 9 am (n = 7, 46). (**D**) Percentages of the embryos are similarly tallied in the reciprocal 129xJF1 parental strain background from the *Ehmt2*^+/−^ x *Ehmt2*^+/−^ cross. The number of pregnant mothers and embryos were: E8: 9 am (n = 1, 8), E8: 1 pm (n = 4, 30), and E8: 6 pm (n = 5, 37). (**E-G**) Maternal effect. (**E**) Images of the *Ehmt2*^mat−/+^ embryos from the *Ehmt2*^fl/fl^; Zp3-cre^Tg/0^ x *Ehmt2*^+/+^ mating at E8: 9 am and E9: 9 am. (**F**) Representative images of MATMUT and MATZYG embryos at E9: 9 am. (n = 5, 14). (**G**) Percentage of MATMUT and MATWT embryos at the somite stages as shown by colors (to the right). E8: 9 am (n = 2, 7) and E9: 9 am (n = 2, 6). (**H**) Parental haploinsufficiency and developmental delay. Tally of *Ehmt2*^+/+^ MATHAPLO embryos from the *Ehmt2*^+/−^ x *Ehmt2*^+/+^ cross (n = 6, 25), and the *Ehmt2*^+/+^ PATHAPLO embryos from the *Ehmt2*^+/+^ x *Ehmt2*^+/−^ cross (n = 4, 30), compared with CONTROL embryos from the *Ehmt2*^+/+^ x *Ehmt2*^+/+^ cross (n = 5, 17) and WT embryos from the *Ehmt2*^+/−^ x *Ehmt2*^+/−^ cross (n = 17, 91).

The development of the mammalian embryo requires chromatin remodeling by the activity of epigenetic modifiers. Maternal mutation experiments reveal whether the availability of a factor in the oocyte is required in the early embryonic stages (Fig 1A, compare cross B versus control cross A) [1–15]. Zygotic mutation experiments (Fig 1A, cross E) reveal whether a factor is needed for development at the late preimplantation, postimplantation and fetal stages [16–18]. In addition, the dose of these epigenetic modifiers in the gametes (parental haploinsufficient) may have lasting effects on the offspring (Fig 1A, cross C versus cross A and cross D versus cross A). For example, a reduced dose of SETDB1, an H3K9 methyltransferase, during male gametogenesis affects gene expression in the offspring [19]. The increased dose of KDM1A, an H3K4 demethylase, during spermatogenesis resulted in reduced H3K4 methylation and severely impaired development and survivability of the offspring and affected its transcriptome [20]. Currently no study exists that integrates these different genetic deficiencies for a specific gene. Potential effect of biparental haploinsufficiency is not considered in genetic experiments when heterozygous intercrosses are used to generate mutant and wild type (biparental haploinsufficient) offspring (Fig 1A Cross E). We designed a mouse study that allows measuring the effect of different deficiencies of the same epigenetic modifier on development and on the genome-wide transcription of the embryo. We included zygotic, maternal, and maternal-zygotic mutant embryos in one comprehensive experiment together with mono-or biparental haploinsufficient wild type individuals and control wild type individuals from wild type parents (Fig 1A). We focused on the deficiencies of the euchromatic histone H3 lysine-9-methyltransferase 2 (*Ehmt2*) gene.

The *Ehmt2* gene encodes EHMT2, the major euchromatin-specific H3K9 histone dimethyltransferase [21], with a complex effect on embryonic development. *Ehmt2*^−/−^ zygotic mutant embryos die around 10.5 days post coitum (dpc) without exception [18,21]. However, unlike the zygotic deficiency, the maternal mutation does not lead to fully penetrant lethality despite a strong maternal phenotype in the majority of the embryos [2]. It is not known whether *Ehmt2* paternal or maternal or biparental haploinsufficiencies have any long-lasting effect on embryo development or transcription. It is not known why some maternal mutants survive but all zygotic mutants die.

To reveal the function of specific regulators, genetic experiments are usually employed to identify differentially expressed (DE) genes between wild type and mutant siblings/uterus mates. Such changes are generally interpreted to be the result of the mutation and are also considered to play a role in the phenotype. One caveat of such experiments is that if the mutation causes a delay in developmental progression, the differences between control and mutant uterus-mate embryos may largely result from comparing two embryos at different developmental stages. Delayed embryo development was reported in *Ehmt2*^−/−^ zygotic mutant embryos between 8.5 and 12.5 dpc [21], at 7.5 dpc [22], and at 6.5 dpc [23]. *Ehmt2*^mat−/+^ maternal mutant embryos show partially penetrant developmental arrest at the 2-cell stage [2]. Those *Ehmt2*^mat−/+^ maternal mutant embryos that pass the 2-cell stage exhibit a mild delay at the 8-cell stage [14]. Variable developmental delay was also detectable in our *Ehmt2* knockout mouse line [24,25], which renders EHMT2 catalytically inactive, and is used in the current study. We hypothesized that delayed development and developmental potential can be connected to changes in the transcriptome.

Understanding how developmental delay is controlled is an exciting question and it requires a different experimental approach beyond comparing uterus-mate mutant and wild type embryos. To investigate the role of EHMT2 in controlling variable developmental delay during embryo development, we collected embryos carrying a series of *Ehmt2* deficiencies (Fig 1A) between 8.5–9.5 dpc and performed total RNA sequencing of somite stage-matched individual embryos. We found differences in the transcriptomes of normal versus deficient embryos and also along normal versus deficient embryo development. We found that EHMT2 plays an important role in reducing transcriptional variation of genes and repeat elements at the six-somite stage embryos, which is consistent with its role in reducing variation in developmental delay.

## Results

### Embryo development shows dose response to zygotic loss of *Ehmt2*

We hypothesized that transcription changes shortly before death of the *Ehmt2* mutations will inform us what causes developmental delay or lethality in the embryos of different deficiencies in the genetic series (Fig 1A). To characterize the developmental consequences of the deficiencies and to find the optimal collection times of somite-stage matched embryos, we first carried out time course experiments.

We examined the *Ehmt2*^*−/−*^ homozygous (HOMO) embryos from the *Ehmt2*^*+/−*^ X *Ehmt2*^*+/−*^ parents (Fig 1A cross E) at different time points and found that they exhibited delayed development compared to their *Ehmt2*^*+/+*^ wild type (WT) and *Ehmt2*^*+/−*^ heterozygous (HET) uterus mates (Fig 1B–1D). Fig 1B displays representative images of uterus-mates with different genotypes. At 6 pm on embryonic day 8, the HOMO embryos only reached the 4 or 6 somite stage when the WT and HET embryos have reached the 12-somite stage. At 9 am on day 9, the HOMO embryos only reached the 12-somite stage while the WT and HET embryos have reached the 24-somite stage. We tallied the results of a larger number of pregnancies at 9 am, 1 pm and 6 pm on day 8, and at 9 am on day 9 of gestation as a percentage of each developmental stage in the total number of embryos collected per time point (Fig 1C). We found that while the majority of WT embryos have reached the 6-somite stage by 9 am on day 8, HOMO embryos reached it at 6 pm on day 8. The majority of WT embryos have reached the 12-somite stage by 6 pm on day 8, whereas none of the HOMO embryos reached this somite number at that time. The embryos shown in Fig 1C were obtained by crossing *Ehmt2*^*+/−*^ mothers in the JF1/Ms genetic background and *Ehmt2*^*+/−*^ fathers in the 129S1 genetic background (Jx1 cross). A reciprocal cross (1xJ) was also set up to validate the developmental phenotype, and to allow us to carry out allele-specific analysis of the transcriptome in the embryos based on SNPs between the mouse strains 129S1 and the JF1/Ms [26] (Fig 1D). We found that the developmental delay of HOMO embryos was reproducible in the reciprocal crosses (Fig 1C and 1D). The tallies revealed an additional observation, which was not apparent at the time of dissections, that the development of HET embryos was slightly slower than the wild type embryos but faster than HOMO suggesting that developmental progression in the embryo was responsive to the dose of EHMT2. Friedman non-parametric statistical tests revealed that the developmental stages of HOMO Jx1 embryos showed significant (*P* = 0.03) difference in median values/variance (Fig 1C), compared WT and HET (*P* = 0.44 and *P* = 0.47, respectively). The HOMO 1xJ and HET 1xJ samples were also significantly variable (*P* = 0.02 and *P* = 0.03, respectively), but the WT 1xJ samples were not (*P* = 0.1) (Fig 1D)

### Embryo development shows dose response to maternal loss of *Ehmt2*

To test the effect of the maternal *Ehmt2* mutation on development, we crossed *Ehmt2*^fl/fl^; Zp3-cre^Tg/0^ females with wild type males. The *Zp3-cre* transgene excises the floxed SET domain-coding exons of *Ehmt2* in the growing oocytes. The resulting maternal mutant *Ehmt2*^mat−/+^ (MATMUT) embryos lack maternal EHMT2 proteins in the oocyte, zygote, 1-cell, and early two-cell stages, but regain zygotic EHMT2 from the normal paternal allele, inherited from the sperm, after the onset of zygotic genome activation. Control *Ehmt2*^fl/+^ embryos (MATWT) were obtained from *Ehmt2*^fl/fl^ mothers in the absence of cre. We found that the MATMUT embryos lagged behind the MATWT embryos, as illustrated in Fig 1E. The delay was even more drastic in maternal-zygotic *Ehmt2*^mat−/−^ (MATZYG) mutant embryos (Fig 1F), which were obtained by crossing *Ehmt2*^fl/fl^; Zp3-cre^Tg/0^ females with *Ehmt2*^*+*/−^ males. The two most advanced MATZYG embryos we recovered had 6 somites or 4 somites (Fig 1F) at 9 am on day 11 of gestation.

We tallied the dissected embryos at 8.5 and 9.5 dpc (Fig 1G). Whereas the mode of somite number was 6 for the MATWT embryos at 9 am on day 8, the MATMUT embryos only reached the 6-somite stage one day later, at 9 am on day 9, when the mode was 24 for the MATWT embryos. We also noticed that the developmental delay of MATMUT embryos varied greatly. At 9 am on day 9 some of these were at the presomite stage but some others displayed 20 somites. Despite the developmental delay observed in the somite-stage embryos, some MATMUT embryos developed to term, reached adulthood, and even reproduced successfully. We obtained live pups by crossing *Ehmt2*^fl/fl^; Zp3-cre^Tg+^ females with males of three different wild type strains and with *Ehmt2*^*+/−*^. The average fecundity rate was 1.7 pups per plug (from 24 plugs total) as compared to 8.5 pups per plug in the control females (S1 Table). This is in agreement with another study that obtained 2.8 MATMUT pups compared to 8.5 control pups [2]. It is noteworthy that the gestation time of the MATMUT embryos was 20.5 days instead of the normal 19.5 days. MATMUT female adults gave birth to an average of 7 pups (from 5 plugs total). This result suggests that the developmental roadblock of MATMUT embryos can be completely overcome in a stochastic manner.

### Embryo development shows dose response to maternal and paternal haploinsufficiency of *Ehmt2*

Next we compared the development of *Ehmt2*^*+/+*^ wild type (WT) embryos derived from two *Ehmt2*^*+/−*^ heterozygous parents to the *Ehmt2*^*+/+*^ wild type (CONTROL) embryos derived from *Ehmt2*^*+/+*^ parents (Fig 1A cross E). The mode was 6 somites in both crosses at 9 am on day 8. While none of the WT embryos has reached the 12-somite stage, 20% of the CONTROL embryos did (Fig 1H). Fewer of the former reached the 8-somite stage also. Because all of these embryos had two normal alleles of the *Ehmt2* gene, the developmental difference may be explained by genetic difference in the parents’ genotype, the WT embryos being biparental haploinsufficient. To further evaluate this possibility, we set up crosses to generate maternal or paternal haploinsufficient *Ehmt2*^*+/+*^ wild type (MATHAPLO or PATHAPLO) embryos from one *Ehmt2*^*+/+*^ parent and one *Ehmt2*^*+/−*^ parent, the mother or the father, respectively (Fig 1A crosses C and D). We tallied the developmental stage of the embryos (Fig 1H) and found that, unlike some CONTROL embryos, none of the MATHAPLO, or PATHAPLO embryos reached the 12-somite stage at 9 am on day 8. This slight delay in both MATHAPLO and PATHAPLO cases, suggests that both the mother’s, and the father’s mutant genotype affected the development of the wild type embryo. This implies that the dose of the EHMT2 protein in the oocyte and the sperm plays a role in instructing developmental programs in the embryo.

### Study design to uncover the role of EHMT2 in preventing variable developmental delay

From the combined results in Fig 1B–1H we can see that the maternal, zygotic and parental haploinsufficient mutations of *Ehmt2* affect developmental progression differently. We hypothesized that the different *Ehmt2* deficiencies (Fig 1A) display unique patterns in their transcriptomes, which would inform us about the specific roles of maternal and zygotic EHMT2 on embryo development, and collected 6-somite and 12-somite stage embryos with the different embryo genotypes, and parental genotypes as listed in S2 Table. The collection times were adjusted to the time course of the mutations. The HOMO embryos, for example, reached the mode of 6-somite stage by 6 pm on day 8, whereas the MATMUT embryos reached it at 9 am at day 9. The 1xJ cross was slightly slower to develop than the Jx1 cross (Fig 1C and 1D). To define the significance of EHMT2 in regulating developmental decisions we also collected WT *and* HOMO embryos at the 12-somite stage, which we will refer to as WT12 and HOMO12 to distinguish them from the 6-somite embryos, WT6 and HOMO6. We prepared total RNA from four replicates of individual embryos, two females and two males, in each condition, except for the very rare maternal-zygotic mutant embryos, which were duplicate females. We carried out allele-specific and strand-specific RNA sequencing analysis on total RNA samples as we did earlier using MEFs [27]. The sequencing depth and data quality is shown in S1 Fig. The homogeneity of the samples is displayed in S2 Fig.

### Three-way comparison of *Ehmt2* zygotic mutant and wild type embryos identifies false positives arising from comparing uterus mates

**Fig 2:**
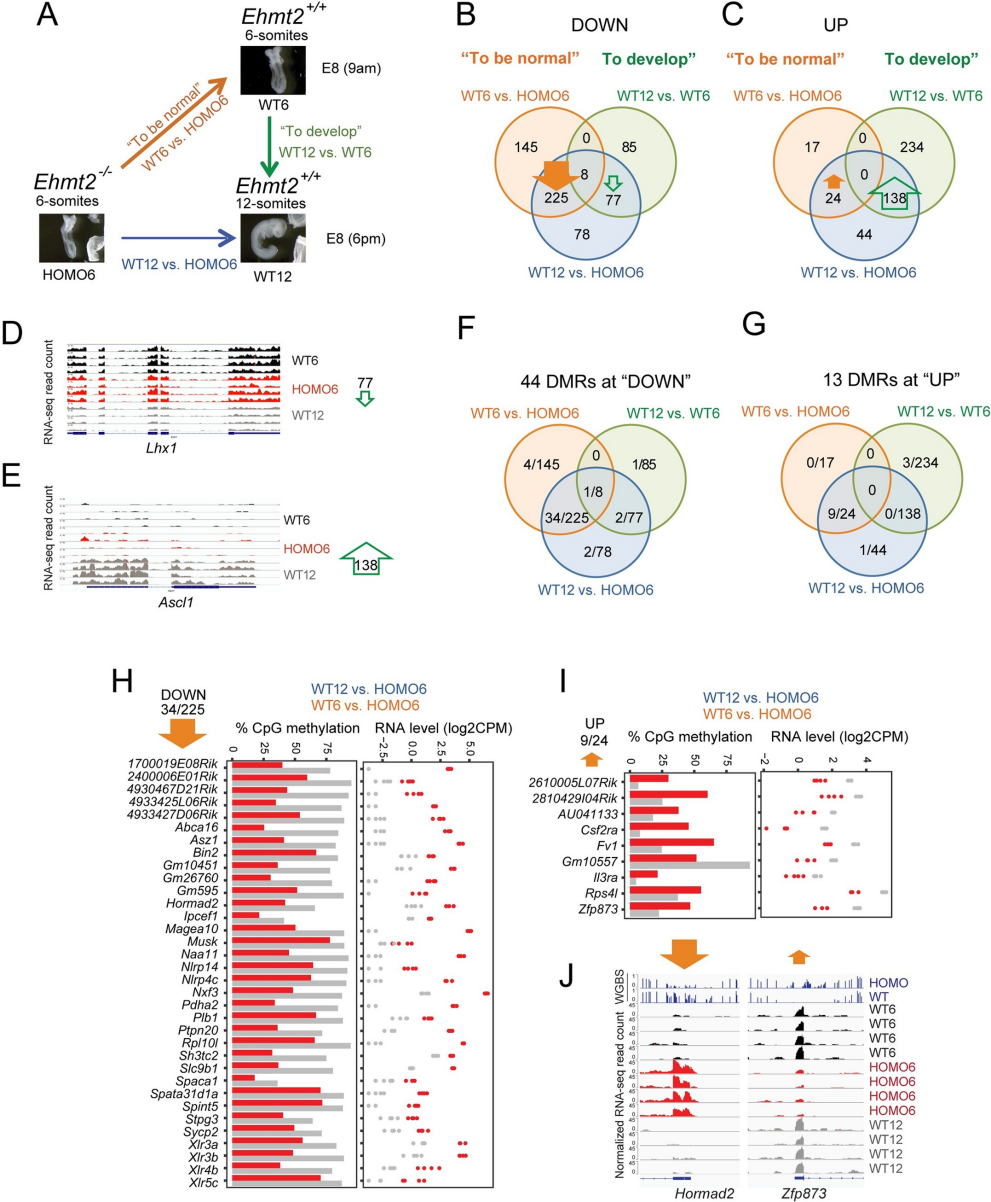
A three-way comparison reveals the direct effects of zygotic *Ehmt2* mutation and identifies false positives of the conventional method that compares siblings. (**A**) Three-way comparison. The conventional comparison (blue arrow) is normally made between uterus-mate mutant and wild type embryos. Because the mutant is developmentally delayed, the differences between these uterus-mates can be divided into two components: 1) “what it takes to be normal” (orange arrow), and 2) “what it takes to develop” from stage one to stage two (green arrow). We illustrate this idea by comparing 6-somite *Ehmt2*^*−/−*^ (HOMO6) and 6-and 12-somite *Ehmt2*^*+/+*^ (WT6 and WT12) embryos. (**B-C**) Results of the RNA-seq experiment are displayed after a 3-way comparison. Venn diagrams depict the number of DEGs along the blue, orange, and green arrows inside the circles of the matching color. (**B**) Genes are downregulated in the condition where the arrow points. EHMT2 is required to suppress 225 genes in WT6 and WT12 embryos (thick arrow). (**C**) Genes are upregulated in the condition where the arrow points. 138 DEGs require EHMT2 according to the WT12-HOMO6 comparison. Because these are also required to reach the 12-somite stage from 6-somite stage in the WT embryos (WT12-WT6 comparison), these DEGs are false positives in the conventional comparison. (**D-E**) IGV browser views are shown of normalized RNA-seq reads at false positive DEGs with reduced (*Lhx1*) or increased (*Ascl1*) expression from WT6 to WT12. **(F-J)** DNA methylation changes occur at a specific subset of DEGs. WGBS analysis in 9.5 dpc WT versus HOMO embryos identified 57 DMRs at the TSS of DEGs. Venn diagrams (**F** and **G**) depict the subset of DEGs, which are differentially methylated. (**H-I**) CpG methylation level (left) and RNA expression level (right) are plotted at DEGs with TSS DMR from the most populated sections of the Venn diagrams displayed in **F** and **G**. Some require EHMT2 for DNA methylation at the TSS and for gene silencing (**H**), others require it for the TSS hypomethylation, and expression (**I**). Grey, WT; red, HOMO. (**J**) IGV browser views are shown of DNA methylation and RNA expression levels at DEGs with reduced (*Hormad2*) or increased (*Zfp873*) DNA methylation in the mutant.

A conventional comparison between uterus-mate HOMO and WT embryos at 8.5 dpc would involve comparing HOMO6 and WT12 embryos according to the modes of somite number at that time (dotted red arrow in Fig 1C). However, such WT12-HOMO6 comparison will result in a mixed outcome and will include two major components as depicted in Fig 2A: 1) “what it takes to be normal” at the 6-somite stage”, which can be assessed by comparing the WT6 versus HOMO6 samples and 2) “what it takes to develop” from the 6- to 12-somite stage, which can be assessed by comparing the WT12 versus WT6 samples. To be able to separate those components, we first performed a three-way comparison of uterus-mate HOMO6 and WT12 embryos, including WT6 embryos in the analysis (Fig 2A). The WT6 embryos had to be obtained from different dams and at different time points (brown arrow in Fig 1C), but they were genetically identical due to the same inbred mouse lines used.

We identified differentially expressed (DE) genes between the three conditions. Each vector (colored arrows in Fig 2A) was assigned a direction toward the more developed and/or the healthier state. We asked what is different between the 12-somite versus 6-somite stage embryos, and what is different between the WT versus HOMO embryos. This latter is unconventional but practical, as it allows intersecting the vectors.

Venn diagrams (Fig 2B and 2C) depict the summary of the 3-way comparison (S3 Table). We detected 388 genes that had decreased expression in WT12 versus HOMO6 embryos. The majority of these hits (225 out of 388) also decreased in the WT6 versus HOMO6 embryos (closed orange arrow in Fig 2B), revealing that those transcripts are suppressed by EHMT2, and belong to the “what it takes to be normal” category. However, 145 DEGs were decreased in the WT6-HOMO6 comparison, and these would be missed in the conventional WT12-HOMO6 comparison. Out of the 388 hits, 77 were found also in the WT12-WT6 comparison (open green arrow in Fig 2B), reflecting developmental changes in transcription that occur between the 6- and 12-somite stages in normal embryos. These changes do not occur in response to the *Ehmt2* mutation but are false positive hits of the WT12-HOMO6 comparison.

We detected 206 genes that showed increased expression in the WT12 versus HOMO6 embryos (Fig 2C). While only 24 out of 206 hits were detected in the intersection with the WT6-HOMO6 comparison (closed orange arrow in Fig 2C), the majority (138 out of 206) occurred in the intersection with the WT12-WT6 comparison (open green arrow in Fig 2C), revealing that those changes occur between the 6- and 12-somite stages during wild type embryo development, independent of EHMT2. These are false positive hits of the WT12-HOMO6 comparison. The false positive hits in Fig 2B and 2C are further illustrated by examples, *Lhx1* and *Ascl1* (Fig 2D and 2E).

### EHMT2 is required for DNA CpG methylation at specific genes in the embryo

In addition to methylating H3K9, EHMT2 also affects transcription by regulating DNA methylation in the embryo and in ES cells at specific genes and transposable elements [23,28–30]. We performed whole genome bisulfite sequencing (WGBS) analysis of HOMO and WT embryo DNA at 9.5 dpc. We searched for differential DNA methylation at the DEGs, which have DNA methylation in either condition at the TSS (+/-1000 bp) (S3 Table). The DMRs most frequently occurred at the intersection of WT12-HOMO6 and WT6-HOMO6 comparisons (Fig 2F and 2G). We plotted the DMR DNA methylation and RNA expression levels of the DEGs from these intersections. DMRs occurred at 34 of 225 DEGs downregulated in the WT samples (Fig 2H), such as *Naa11*, *Asz1*, *Pdha2*, and *Magea10*, *Hormad2*. In each case a higher level of DNA methylation corresponded to less RNA expression in the WT versus HOMO embryo, suggesting that EHMT2-dependent suppression at these genes involves DNA methylation in the WT embryos. On the other hand, 9 DMRs at 24 upregulated DEGs showed a lower level of DNA methylation which corresponded to higher RNA expression in the WT versus HOMO embryos (Fig 2I), suggesting that EHMT2 helps to maintain the DNA hypomethylated and expressed state of these 9 genes in the WT embryo. Examples of the above two classes are displayed by IGV browser images of *Hormad2* and *Zfp873* (Fig 2J). We identified differentially methylated regions (DMR) between the WT and HOMO samples genome-wide (S4 Table) and depicted the distribution of those in pie charts (S3 Fig). DNA methylation required EHMT2 in WT embryos at 305 DMRs (S3A Fig), mostly at intergenic, intronic, and exon locations, whereas DNA hypomethylation required EHMT2 in WT embryos at 89 DMRs (S3B Fig), mostly exons, introns, and promoters. Only a small fraction affected gene expression. Among DMRs that required EHMT2 for DNA methylation 42% of the DMRs that were located in the promoter or 25% of DMRs located in an intron of the nearest transcript affected the level of its transcription (S4 Table). The WGBS analysis in combination with the three-way comparison of RNA-seq results allowed us to conclude that EHMT2–dependent DNA methylation status was more frequent at the DEGs in the “what it takes to be normal” than in the “what it takes to develop” category (50 versus 9 DEGs, respectively).

### Four-way comparison identifies EHMT2-dependent developmental changes in the transcriptome

**Fig 3:**
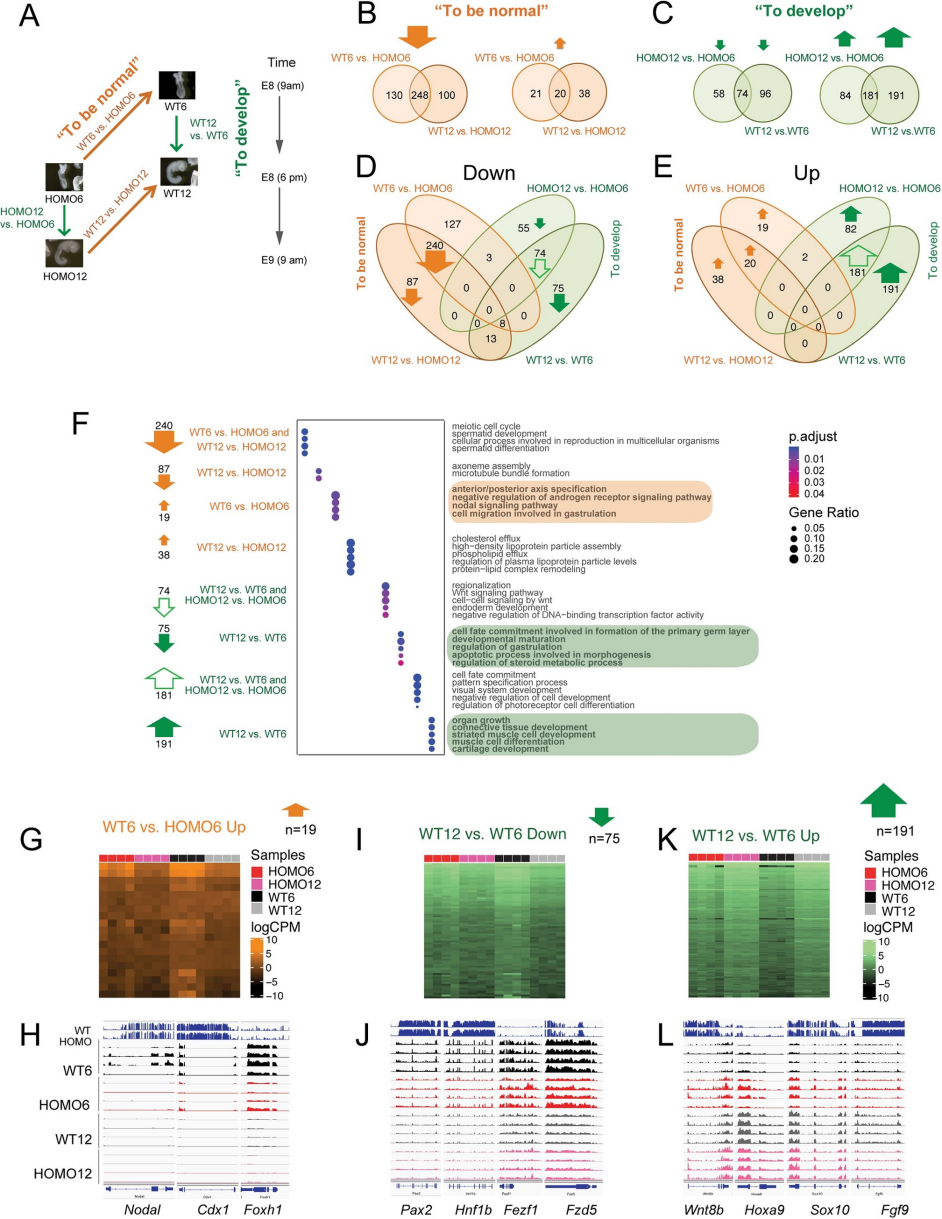
A four-way comparison identifies DEGs that distinguish normal embryo development from *Ehmt2* zygotic mutant embryo development. (**A**) Four-way comparison. The four questions are depicted by arrows: “what it takes to develop” from 6- to 12-somite stage in the normal or in the mutant embryo (green arrows indicate the WT12-WT6 and HOMO12-HOMO6 comparisons), and “what it takes to be normal” at 6-somites or at the 12-somite stage (orange arrows mark the WT6-HOMO6 and WT12-HOMO12 comparisons). (**B**) Identifying what it takes to be normal. Venn diagrams show the number of the downregulated (left) and upregulated (right) DEGs between the *Ehmt2*^*+/+*^ (WT) and the *Ehmt2*^*−/−*^ (HOMO) embryos at the 6-somite stage and at the 12-somite stage. (**C**) Identifying what it takes to develop from 6- to 12-somite stage. Venn diagrams show the number of downregulated (left) and upregulated (right) DEGs between the 12-somite and 6-somite stages, during normal *Ehmt2*^*+/+*^ (WT), or mutant *Ehmt2*^*−/−*^ (HOMO) development. (**D-E**) Venn diagrams merge all of the downregulated (**D**), or all of the upregulated (**E**) DEGs from the two-way comparisons as described above. (**F**) Gene ontology analysis of the DEGs. Bubble plots depict the GO terms enriched in the segments of the 4-way Venn diagrams as indicated to the left and marked by arrows as above. (**G**-**H**) Understanding what it takes to be normal between WT6 vs. HOMO6, but not between WT12 vs. HOMO12 embryos. The Venn diagram segment is displayed in a heatmap (**G**) and as IGV browser examples (**H**). (**I**-**L**) Understanding what it takes to develop. Downregulation of 75 genes (**I**) or upregulation of 191 genes (**K**) only occurs between the 6-and 12- somite stages only in normal development (WT12 vs. WT6), but not in mutant development (HOMO12 vs. HOMO6); therefore, these developmental-specific changes require EHMT2. These are false negatives of the conventional WT12-HOMO6 comparison. Heatmaps (**I** and **K**) are shown for the Venn-diagram sections and IGV browser examples (**J** and **L**) are shown for the DEGs that drive the GO term in those sections.

To further improve the interpretation of our RNAseq experiment we included one more condition, the 12-somite stage *Ehmt2*^*−/−*^ embryo (HOMO12) in our analysis. This four-way comparison (Fig 3A) has two advantages over the three-way comparison: we can now also determine “what it takes to be normal” in the embryo at the 12-somite stage by contrasting WT12-HOMO12 and “what it takes to develop” normally or abnormally between the 6- and 12 somite stages by using the contrasts of WT12-WT6 and HOMO12-HOMO6 embryos. As in the three-way comparisons, asking what is different between the 12-somite versus 6-somite stage embryos, and what is different between the WT versus HOMO embryos is unconventional, but allows us to intersect the vectors.

To find out what it takes to be normal, we identified 378 downregulated DEGs in the WT embryos using comparisons of the WT6-HOMO6 and 348 between WT12-HOMO12 (S5 Table and Fig 3B). 248 of these DEGs overlapped between the two contrasts. Compared to 478 downregulated genes, much fewer, only 79 DEGs were upregulated in the WT embryos in the totality of WT-HOMO comparisons. To find out what it takes to develop, we identified 372 and 265 upregulated DEGs at the 12-somite stage in the WT12-WT6 and HOMO12-HOMO6 contrasts, respectively. 181 DEGs sorted into the intersection (S5 Table and Fig 3C). Compared to 456 upregulated genes, only 228 were downregulated in the totality of the 12-somite versus 6-somite comparisons. We found that “what it takes to be normal” are mainly DEGs that require EHMT2 for suppression (Fig 3B), and “what it takes to develop” are DEGs that require EHMT2 for their activation along development (Fig 3C).

We merged the Venn diagrams into downregulated (Fig 3D), or upregulated (Fig 3E) DEGs from the two-way comparisons. It was interesting to note that, with very few exceptions, the DEGs populated the 6 compartments on the outside of the composite Venn diagrams, revealing that there is very little overlap between the DEGs that belong to “what it takes to be normal” at the left side and “what it takes to develop” to the right of each Venn. The diagrams illustrate also that the majority of DEGs are either downregulated in WT versus HOMO embryos (Fig 3D) or upregulated in 12-somite versus 6-somite stage embryos (Fig 3E). We performed a pathway analysis (Fig 3F, and S6 Table) of the DEGs from the prominent sections of the Venn diagrams. We also generated heatmaps and browser examples from these transcript sets (Fig 3G–3L and S4F Fig). First, we looked at the “what it takes to be normal” categories. The intersection of WT6-HOMO6 and WT12-HOMO12 contain 240 downregulated DEGs (Figs 3D, S4A and S4B) that require EHMT2 for their suppression in WT embryos regardless of somite number of 6 or 12. These DEGs are enriched in gene ontology (GO) terms, such as meiotic cell cycle, spermatid development and differentiation, and cellular process involved in reproduction (Fig 3F), including *Asz1*, *Dnmt3l*, *Sycp2*, *Rnf212*, *Syce1*, *Piwil4*, *Hormad2*, *Smcb1*, *Spaca1*, *Cftr*, *Zpbp*, *Ccdc42*, and the *Xlr3-Xlr5* cluster (*Xlr4c*, *Xlr3a*, *Xlr3c*, *Xlr3b*, *Xlr4a*, *Xlr4b*, *Xlr5a*, *Xlr5c*, and *Xlr5b*). The 20 genes upregulated in both the WT6-HOMO6 and WT12-HOMO12 comparisons (Figs 3E, S4C and S4D) include DEGs, such as *Sycp1*, *Tssk6*, *Il3ra*, and *Csf2ra* and GO terms, such as sperm chromatin condensation, spermatid nucleus differentiation, reciprocal meiotic recombination, homologous recombination, and synapsis (Fig 3F and S6 Table).

The WT12-HOMO12 ‘Down’ compartment includes 87 genes downregulated in WT12 such as *Dnah1a*, and *Dnah1b* (S5 Table and Fig 3D), and GO terms, such as axoneme assembly and microtubule bundle formation (Fig 3F). The WT12-HOMO12 ‘Up’ compartment includes 38 genes upregulated in WT12 (S5 Table and Fig 3E), such as *Apoa1*, *Apoa4*, *Apoc2* and *Apom* and terms related to protein-lipid complex remodeling (Fig 3F). The WT6-HOMO6 ‘down’ genes had no significant GO terms. We found 19 genes in the WT6-HOMO6 ‘Up’ compartment, such as *Lefty1*, *Nodal*, *Cdx1*, and *Foxh1* (Fig 3E, Fig 3G and 3H), which belong to developmental processes, such as anterior-posterior axis specification, negative regulation of androgen signaling pathway, nodal signaling, and cell migration involved in gastrulation (Fig 3F and S6 Table).

Next, we inspected the pathways associated with DEGs in the “what it takes to develop” categories. The intersections of WT12-WT6 and HOMO12-HOMO6 comparisons inform us what it takes to develop from 6-to 12-somite stage in the WT and also in the mutant embryos, undisturbed by the mutation. We found 74 DEGs in the intersection that are suppressed in the 12-somite stage compared to the 6-somite conditions (Figs 3D, S4E and S4F), such as *Pou5f1*, *Cfc1*, *Zic3*, *Ar*, *Eomes*, *Gsc*, *Foxh1*, *Wnt8a*, *Cdx1*, *Amer3*, *Notum*, *Lect2*, *Sfrp5*, *Phlda2*, *Hesx1*, and *Pgr*. The GO terms, such as regionalization, Wnt signaling pathway, and endoderm development were enriched (Fig 3F). We found 181 DEGs in the intersection that are upregulated in the 12-somite stage compared to the 6-somite conditions (Figs 3E, S4G and S4H), such as *Hoxa10*, *Hoxa9*, *Hoxa10*, *Hoxd10*, *Hoxd11*, *Lhx6*, *Dlx1*, *Dlx2*, *Neurod1*, *Neurod4*, *Neurog1*, *Myl2*, *Cyp26b1*, *Dbx1*, *Nr2f1*, *Nr2f2*, *Nr2e1*, *Sostdc1*, *Gdf7*, *Gdnf*, *Nkx2-1*, *Nkx3-2*, *Olig2*, *Sox8*, *Meox2*, *Pax1*, *Alx4*, *Uncx*, *Onecut1*, and *Ascl1*. These DEGs were associated with GO terms, such as pattern specification, cell fate commitment, and visual system development (Fig 3F and S6 Table).

The HOMO12-HOMO6 (but not WT12-WT6) compartments of the Venn diagram revealed 55 downregulated and 82 upregulated DEGs (Fig 3D and 3E) that are specific to mutant embryo development. These DEGs did not result in any significantly enriched GO term. The WT12-WT6 (but not HOMO12-HOMO6) compartments of the Venn diagram revealed 75 downregulated and 191 upregulated DEGs (Fig 3D and 3E) that are specific to normal embryo development. The 75 DEGs genes downregulated in WT12 (Fig 3D, 3I and 3J) included *Pax2*, *Fzd5*, *Hnf1b*, *Fezf1*, *Nanog*, *Nodal*, and *Mesp1*, and GO terms such as cell fate commitment involved in formation of the primary germ layer, developmental maturation, regulation of gastrulation, and apoptotic process involved in gastrulation (Fig 3F and S6 Table). The 191 DEGs upregulated in WT12 (Fig 3E, Fig 3K and 3L) included *Erbb4*, *Neurog2*, *Wnt7b*, *Wnt8b*, *Myf5*, and *Fgf9*, and the GO terms such as organ growth, connective tissue development, striated muscle development, muscle differentiation, and cartilage development (Fig 3F and S6 Table). The WT12-WT6 compartment of DEGs is the most interesting as it reveals developmental changes in the transcriptome that require EHMT2.

### EHMT2 suppresses variation of DEGs at the 6-somite stage

Looking at the heatmap displays (Fig 3I and 3K) of WT12-WT6 DEGs more closely we noticed that downregulation or upregulation of the DEGs did take place from the 6-to 12-somite stages in both WT and HOMO embryos even though we could only call the significant changes in the WT12-WT6 but not in HOMO12-HOMO6 comparisons. Interestingly, the HOMO6 embryos displayed a high level of variation (Fig 3I–3L), which could explain this discrepancy. We showed earlier that the samples are high-quality across the metrics of library size, duplication rate, mean sequence quality, sequence length and alignment rate (S1 Fig), and are homogeneous (S2A Fig) suggesting that sequence quality cannot explain the observed expression variability. The variation was mainly inter-embryo and the individual HOMO6 embryos showed global differences in the genes that “takes to develop normally” (Fig 3I and 3K). For example, embryo 2 showed globally reduced levels of the gene set in the ‘Down’ category but globally increased levels in the ‘Up’ category, therefore, it was more similar to WT12 embryos. Embryo 4 on the other hand, was more similar to WT6 embryos. Embryos 1 and 3 were in between those extremes. We concluded that in the WT12-WT6 compartment the high variation in the HOMO6 samples prevented us from calling statistically significant developmental changes in the HOMO12-HOMO6 comparison.

**Fig 4:**
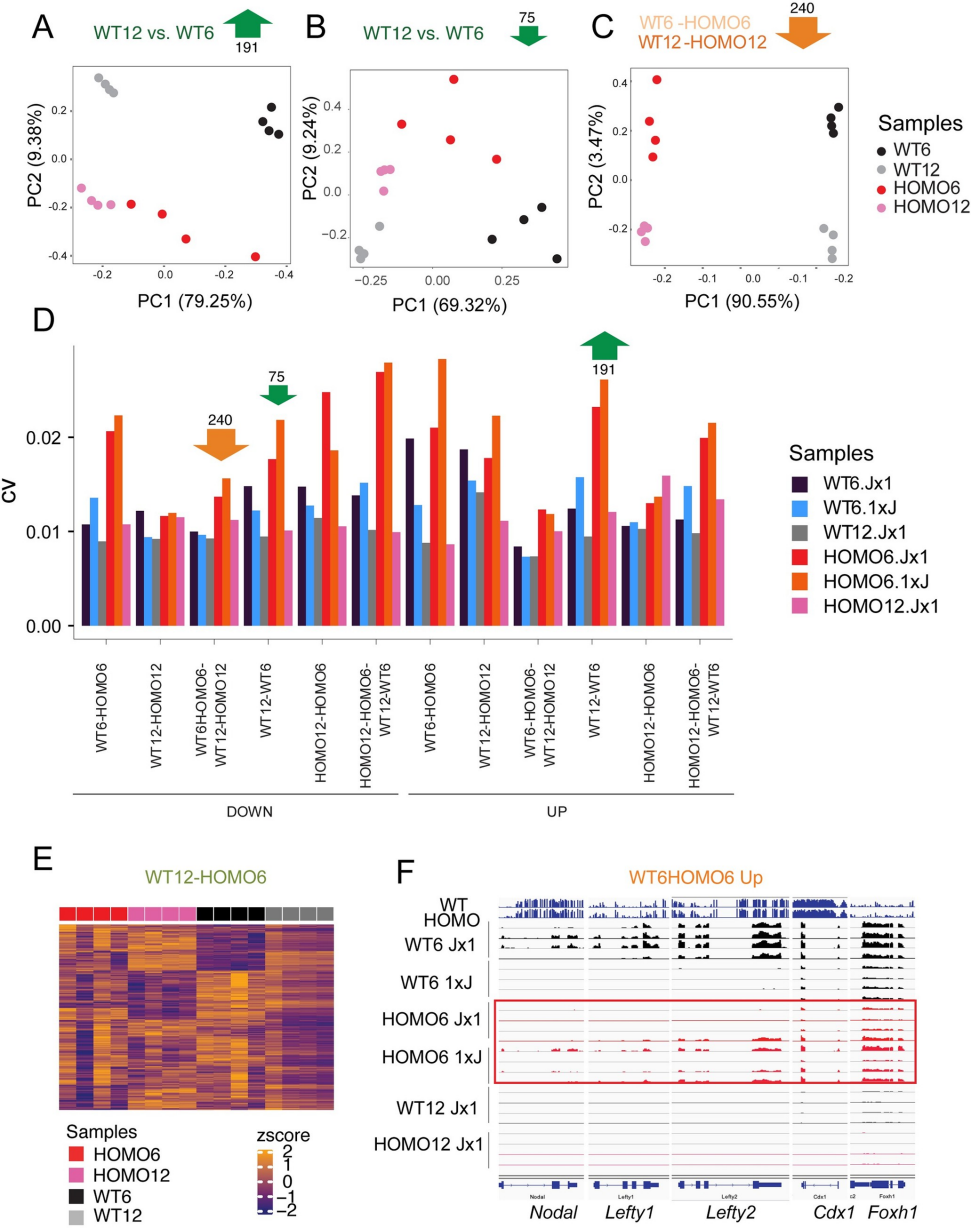
*Ehmt2* zygotic mutant embryos display an increased transcriptional variation. (**A-C**) Principal component analysis is shown for the samples marked to the right based on the DEGs derived from the four-way comparison as indicated at the top. (**D**) Bar graph displays the coefficient of variation in the samples (as color-coded to the right) in each comparison (as marked at the bottom). The HOMO6 and WT6 samples were analyzed in both the Jx1 and reciprocal 1xJ crosses. Note that the HOMO6 samples are the most variable. (**E**) Heatmap shows the z-scores of the genes that display the highest level of dispersion in the WT12-HOMO6 comparison based on MDSeq analysis. The HOMO6 samples are the most dispersed. (**F**) Variation is observed in a group of DEGs that belong to the WT6-HOMO6 comparison. IGV browser images of embryo samples are shown, four replicates in each condition as marked. The top two lanes are WGBS sequencing results.

To explore EHMT2-dependent variation in more detail, we performed a principal component analysis (PCA) of the upregulated 191 DEGs and downregulated 75 genes (Fig 4A and 4B) in the WT12-WT6 compartment, as defined in the Venn diagrams (Fig 3D and 3E). Whereas WT6, WT12, HOMO12 samples were tightly clustered in the first and second principal components, the HOMO6 samples were scattered in both directions. In contrast, we did not find such scattering of the HOMO6 samples in the PCA plot of the downregulated 240 DEGs (Fig 4C) from the intersection of WT6-HOMO6 and WT12-HOMO12 comparisons (Fig 3D). This suggests, that EHMT2 more strongly reduces the variation of DEGs, which “takes to develop normally” than those, which “takes to be normal”. We calculated the coefficient of variation for each sample in the sections of the 4-way Venn diagrams (from Fig 3D and 3E) and found that the HOMO6 samples exhibited the greatest variability in 9 out of 12 comparisons (Fig 4D). This was true for the HOMO6 samples obtained in both the Jx1 and 1xJ crosses. In addition, Using MDSeq [31], we identified a set of genes, which are significantly (FDR < 0.05) differentially variable between the WT12 and HOMO6 embryos. The heatmap (Fig 4E) shows the Z-scores of those genes in the HOMO6, HOMO12, WT6 and WT12 embryos. Again, the HOMO6 embryos displayed the most heterogeneity. We showed earlier that the HOMO6 samples were the most variable among DEGs of the WT12-WT6 comparisons (Fig 3I and 3K). In addition, the HOMO6 samples were variable in the group of developmental genes, such as *Nodal*, *Lefty1*, and *Foxh1*, which were downregulated from the 6- to the 12-somite stage embryos but also belonged to the WT6-HOMO6 (Jx1) upregulated category (S5 Table). We found that, even though these genes exhibited tight regulation in WT6 embryos, albeit at different levels of expression, they showed variability in the HOMO6 Jx1 and 1xJ embryos (Fig 4F). In summary, our results suggest that EHMT2 suppresses the variation of developmental gene expression at the 6-somite stage.

### Transcriptomes of the *Ehmt2* maternal-zygotic mutant embryos resemble zygotic mutants, and the transcriptomes of parental haploinsufficient wild type embryos are normal

**Fig 5:**
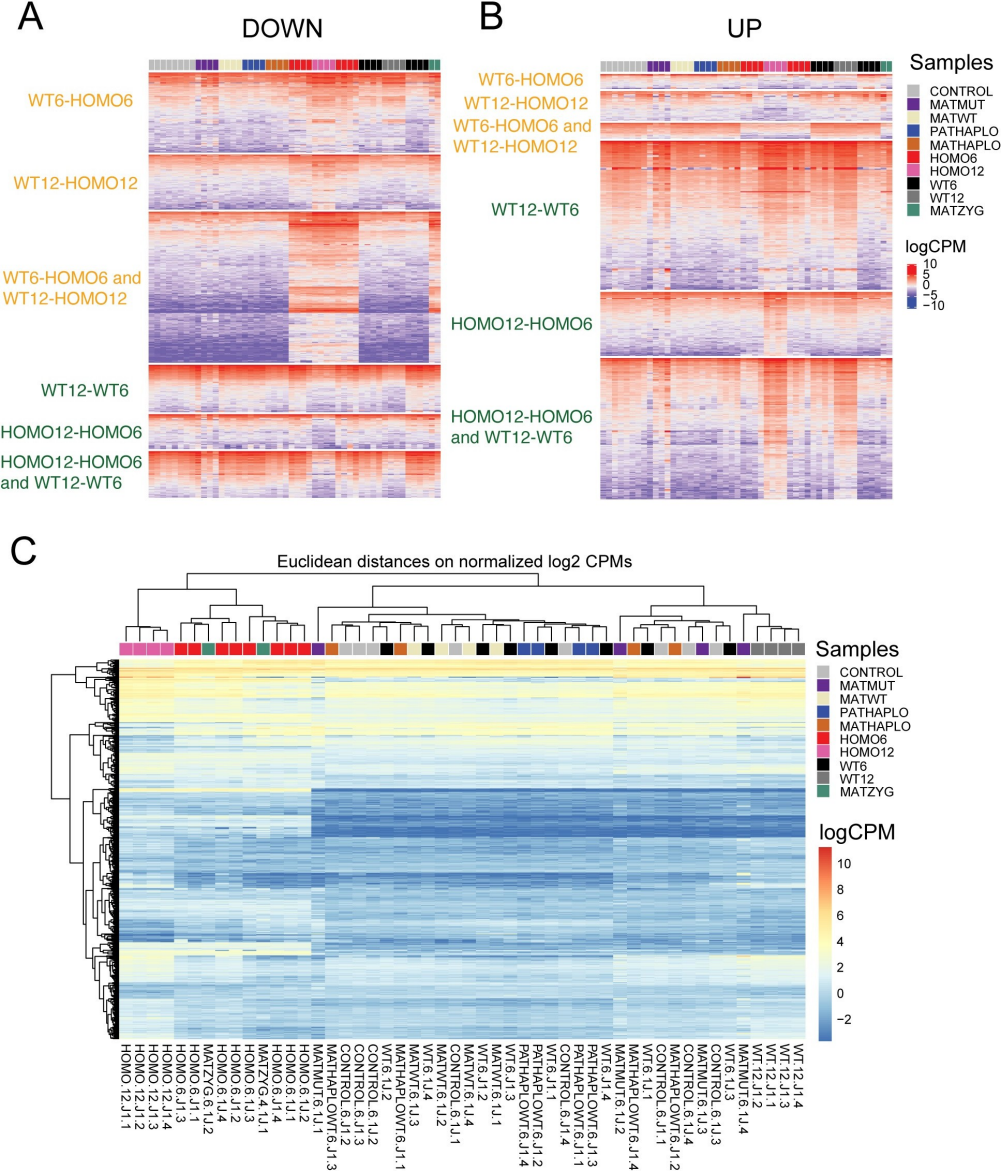
Transcriptomes of embryos with different *Ehmt2* genetic deficiencies. Heatmaps display gene expression values (logCPM) for the downregulated (**A**) and upregulated (**B**) genes from the compartments of the Venn diagrams in Fig 3D and 3E, as marked, representing the full dataset (Fig 1A and S2 Table). Four replicates are shown in the samples coded by color (to the right). (**C**) Heatmap displays the results of an unsupervised cluster analysis that calculates Eucledian distance between samples based on the DE genes identified in the four-way comparison displayed in the Venn diagrams in Fig 3D and 3E.

After characterizing the zygotic mutant embryos, we generated heatmaps (Fig 5A and 5B) that show the transcriptional profile of each transcript out of the 6 prominent compartments of the Venn diagrams (Fig 3D and 3E) across each sample of our study in the entire dataset (S2 Table). The categories of “what it takes to be normal” are seen at the top and the categories “what it takes to develop” are seen at the bottom. The MATMUT6 embryos displayed a highly variable pattern and resembled 12-somite embryos in the developmental comparisons (Fig 5A and 5B). The transcriptome of MATZYG mutant embryos was very similar to the HOMO embryos in the WT6-HOMO6 and WT12-HOMO12 contrasts while it resembled the 6-somite embryo in the WT12-WT6 and HOMO12-HOMO6 contrasts (Fig 5A and 5B). Overall, the zygotic mutation dominated the transcription pattern over the maternal mutation in these rare MATZYG embryos. We also performed an unsupervised hierarchical clustering of the sample set based on the DE genes of the 4-way comparison and found that the samples are divided based on zygotic mutation. The 12-somite samples were in distinct subclusters in the two major clusters. MATZYG samples clustered with HOMO samples (Figs 5C and S2B). However, MATHAPLO, PATHAPLO and MATHET samples clustered with WT and CONTROL samples (Fig 5C).

We identified DE genes using the relevant comparisons. The MATHAPLO, PATHAPLO and WT (biparental haploinsufficient) samples only differed from the CONTROL samples, at a total 8, 7, and 4 DEGs (S5 Table) and these did not provide GO enrichment. We can say that the transcriptomes of all parental haploinsufficient embryos were normal, in agreement with the full potential of those embryos to develop to term and reproduce despite the initial slight developmental delay (Fig 1H).

### Maternal EHMT2 suppresses transcriptional variation in the embryos

**Fig 6:**
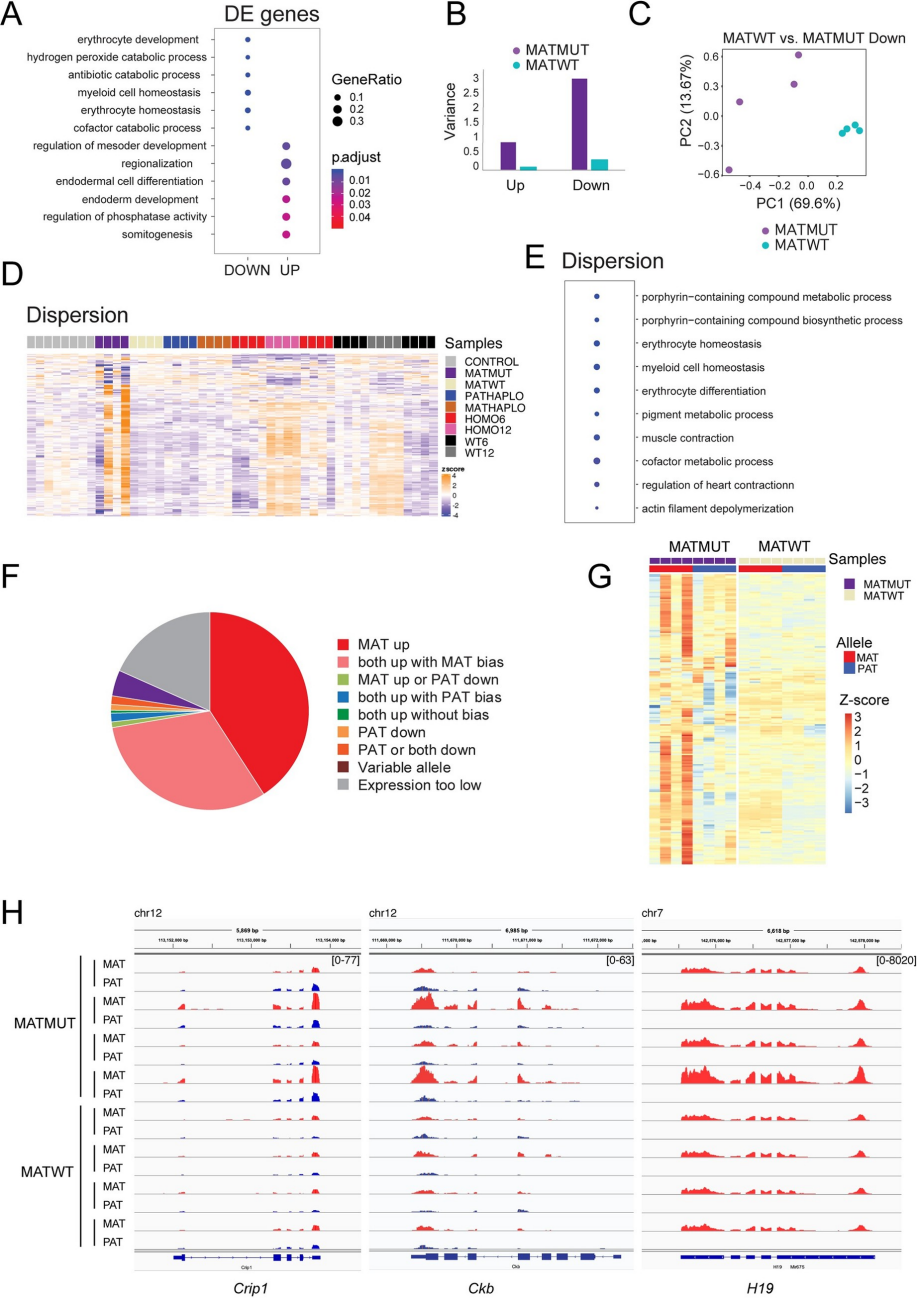
Maternal *Ehmt2* mutation results in an increased transcriptional variation. (**A**) Bubble plot from gene ontology analysis shows the most significantly enriched biological processes derived from the DEGs of MATZYG embryos compared to MATWT embryos. (**B**) Bar graph depicts the variance of DEGs identified between *Ehmt2*^mat−/+^ (MATMUT) embryos and control (MATWT) embryos at the 6-somite stage. (**C**) Principal component analysis of the DEGs downregulated in MATWT compared to MATMUT embryos. (**D**) Heatmap of the 500 genes that exhibit the highest level of dispersion in the MATMUT-MATWT comparison. The z-scores are displayed across the full set of samples. Note that most of these genes that are variable in MATMUT embryos show variation also between the 6- and 12-somite stages. (**E**) Gene ontology analysis depicts the biological processes derived from the most variable genes in MATMUT embryos. (**F**) The maternal allele is dominant in the highly variable transcripts in MATMUT embryos. Pie chart depicts the classes that are based on the proportion of changes that occur in the different parental alleles. (**G**) Heatmap displays that the variability of MATMUT transcripts is mainly in the maternally inherited allele. We obtained the variance stabilization transformed reads then plotted the gene-wise Z-scores for the 197 most variable genes in replicates of MATMUT and MATWT embryos. (**H**) IGV browser images are shown of examples for highly variable transcripts in the top classes. In MATMUT embryos *Crip1* is derepressed in both alleles, but the maternal allele is more derepressed than the paternal allele (both up with MAT bias), *Ckb* is derepressed in the maternal allele while the paternal allele does not change, and *H19* is derepressed in the maternal allele while the paternal allele stays silent (MAT up).

To reveal the long-lasting effect of maternal EHMT2 depletion in the oocyte on the transcription regulation in postimplantation development, we compared MATMUT and control MATWT embryos at the 6-somite stage. A total of 190 DEGs were identified in this comparison (S5 Table). These DEGs were enriched in GO terms including erythrocyte development and homeostasis, myeloid cell homeostasis, regulation of mesoderm development, regionalization, endodermal cell differentiation, somite development (Fig 6A and S7 Table). The variability of MATMUT samples observed in the heatmaps (Fig 5), prompted us to quantify variation in the MATMUT embryos. We calculated the average variance across genotypes and found that the variance was greater in the MATMUT embryos compared to the MATWT controls among both upregulated and downregulated DEGs (Fig 6B). The PCA for the downregulated genes (Fig 6C) showed greater scattering of the MATMUT samples than the MATWT samples in the first two principal components. Using MDSeq, we identified the set of genes, which were significantly (FDR < 0.05) differentially variable between the MATMUT and MATWT control embryos (S8 Table). The heatmap (Fig 6D) shows the Z-scores of the differentially variable genes in the MATMUT, and MATWT, embryos, among other samples of the current analysis. We performed a GO analysis on the set of differentially variable genes and displayed the ten most significant terms in the bubble plot (Fig 6E). Interestingly, the pathways again included erythrocyte homeostasis and differentiation and myeloid cell homeostasis (S7 Table). The most dispersed transcripts in the MATMUT embryos appeared to change developmentally between the 6-somite and 12-somite stages irrespective of mutant status (Fig 6D). This tells us that the maternal mutation increases the variation at a set of genes that change during normal development. We asked whether the increased transcriptional variation in MATMUT embryos occurred in the maternal allele. We found that the maternal allele was derepressed exclusively in 41% of those highly variable changes including imprinted genes, and in another 31% of those changes both alleles were derepressed, but the maternal allele was dominant (Fig 6F and 6G and S8 Table). We display examples in Figs 6H and S5. The high variability of transcription in the maternal allele is consistent with a disturbance during MATMUT oogenesis due to the absence of EHMT2.

### EHMT2 regulates the expression and variability of transposable elements

**Fig 7:**
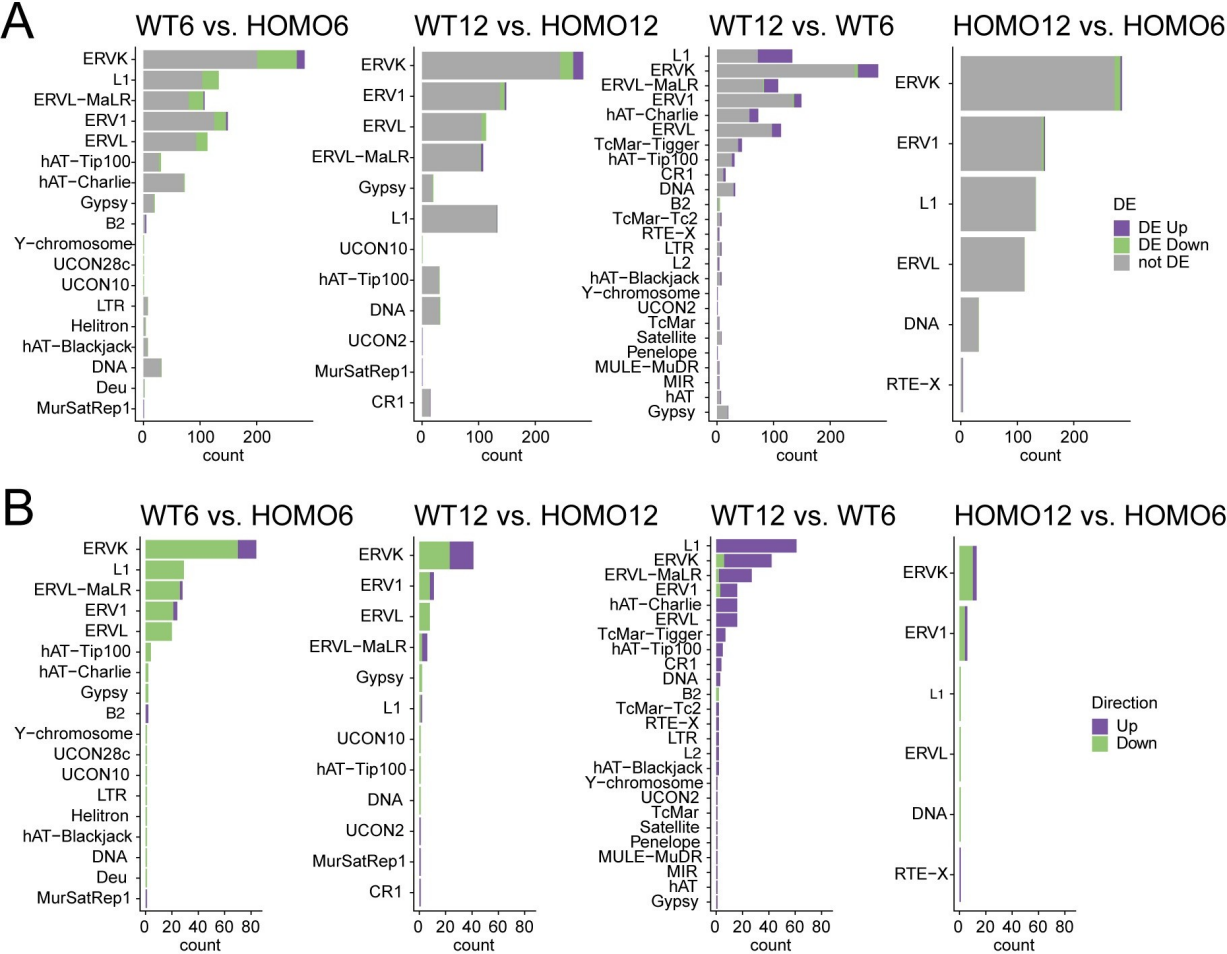
EHMT2 regulates transposable element subfamilies. (**A**) Bar charts depict the number of TE subfamilies that are up- or downregulated by EHMT2 in a given TE family (as written to the left) that shows changes in the contrasts indicated at the top. (**B**) Bar charts depict the number of TE subfamilies that are up- or downregulated by EHMT2 in a given TE family (as written to the left) that shows changes in the contrasts indicated at the top.

It is known that H3K9 methylation is required for suppressing transposable elements (TEs) in the genome. Such suppression has been shown in the male germ line and in ES cells [23,28,30,32,33]. We asked whether EHMT2 regulates TEs in the embryos at 6-and 12-somite stages. We first analyzed the relative expression of TE subfamilies. We plotted the number of TE subfamilies that are up- or downregulated by EHMT2 in a given TE family that shows changes in the four-way comparison (S9 Table and Fig 7A and 7B). LTR class subfamilies that belong to the ERVK, ERVL-MaLR, ERV1, and ERVL families and the L1 LINE elements were the most numerous DE repeats between WT6-HOMO6 and WT12-HOM12 embryos. This was in agreement with the findings in 6.5 dpc *Ehmt2*^−/−^ zygotic mutant epiblasts [22]. We found that endogenous retroviral element (ERV) subfamilies were mainly suppressed by EHMT2 in WT6 embryos according to our WT6-HOMO6 comparison, they were developmentally derepressed according to the WT12-WT6 comparison (Fig 7).

LTRs of class III retrotransposons ERVL-MaLR and ERVL serve as alternative promoter for initiating transcription specifically in 2-cell embryos at the time zygotic genome activation, and oocyte-specific transcripts initiate from ERVL-MaLR and ERVK LTRs during oocyte development [34–36]. Suppression of the 2-cell specific LTRs is associated with reduced H3K9me2, at least in ES cells [37] where suppression of class III ERVL LTRs depends on the catalytic activity of EHMT2 [33]. We found that the 2-cell and oocyte-specific RTL-driven chimeric transcripts (including *Spin1*, *Dnajc11*, Vdac2, *Nfil3*, *Pard3*, *Calr3*, *Abcb1b*, *Stk3*, *Ski*, *Zfp352*, *Zfp277*, *Eif1a*, *Dub1a*, *Slfn4*, *Gm10696*, *Tmem56*, *Tmem92*, *Tdpoz3-4*, *Dcc*, *Arg2*, *Plagl1*, *Impact*, *Slc38a4*) do not become derepressed in HOMO6 and HOMO12 embryos.

**Fig 8:**
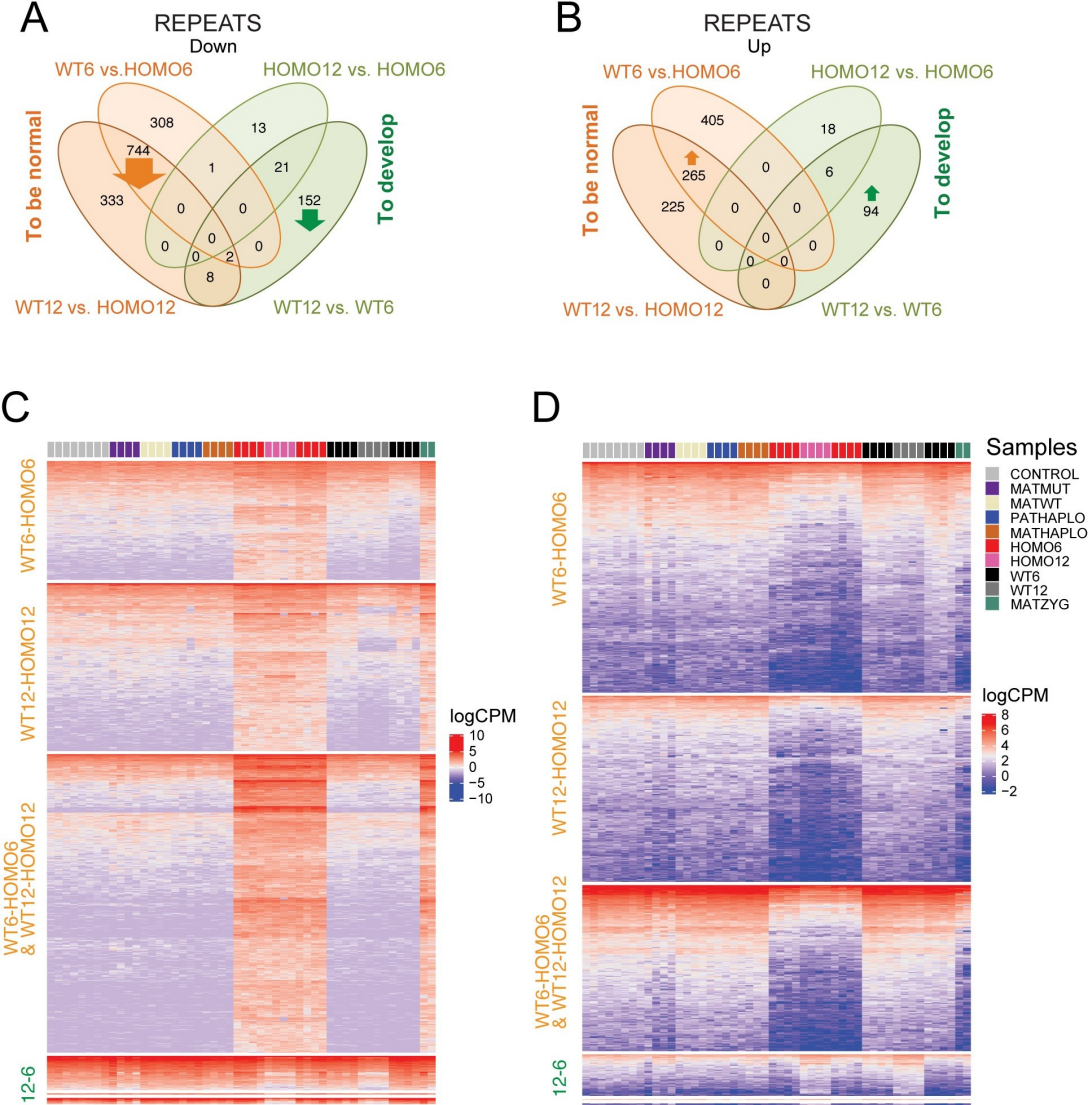
Four-way comparison of uniquely mapped transposable elements. (**A-B**) Venn diagram showing downregulated (**A**) and upregulated (**B**) repeats which were identified using uniquely mapped reads and were stratified according to the four-way comparison. (**C-D**) Heatmaps of downregulated (**C**) and upregulated (**D**) repeats as shown in the above Venn diagrams, with comparisons labeled to the left, samples indicated at the top and color codes shown to the right. The category labeled 12–6 contains WT12-WT6 and HOMO12-HOMO6 and their intersection. Note that most DE repeats are related to genotype. Note the variability of derepression in HOMO6 and MATMUT samples. Note that MATZYG embryos are most similar to HOMO6 embryos.

We identified differentially expressed (DE) repeats using uniquely mapped reads in our datasets and classified the DE repeats according to our four-way comparison (Fig 8A and 8B and S10 Table). We found that most DE repeats belong to the “what it takes to be normal” category. Repeats are downregulated (Fig 8A and 8C) or upregulated (Fig 8B and 8D) in the WT versus HOMO embryos. Specifically, 744 repeats showed downregulation in the intersection of WT12-HOMO12 and WT6-HOMO6 comparisons (orange arrow in Fig 8A), suggesting that these were suppressed by EHMT2 in the WT condition, irrespective of somite number. These belonged to many different repeat classes (S6A Fig). Unexpectedly, 265 TEs were upregulated in the intersection of WT12-HOMO12 and WT6-HOMO6 comparisons (orange arrow in Fig 8B), suggesting that these elements require EHMT2 for activation in the WT condition irrespective of somite number. These 265 repeats belonged predominantly to the LTR class (S6B Fig). Furthermore, 333 TEs showed downregulation in the WT12-HOMO12 but not WT6-HOMO6 comparison, and 308 repeats showed the opposite (Figs 8A and S6C). Also, 225 TEs showed upregulation in the WT12-HOMO12 but not WT6-HOMO6 comparison, and 405 repeats showed the opposite (Fig 8B). SINE elements were frequently found in these 405 repeats (S6D Fig).

We next identified the DE repeats that belonged to the “what it takes to develop” category (Fig 8 and S10 Table). We found that 152 repeats (mostly SINEs located at intergenic regions) were suppressed and 94 repeats (mostly LINEs) were activated in WT12 compared to the WT6 condition (S6E and S6F Fig). It was interesting to note that the WT12-WT6 DE repeats displayed the greatest variation at the 6-somite stage, similar to WT12-WT6 DE genes (Fig 8D). This suggests that EHMT2 regulates variation not only of genes but also of TEs.

We noticed that similar to DE genes, the DE repeat profile of MATZYG mutant embryos resembled HOMO embryos in the WT6-HOMO6 and WT12-HOMO12 contrasts while it resembled the 6-somite embryo in the WT12-WT6 and HOMO12-HOMO6 contrasts (Fig 8C and 8D). Overall, the zygotic mutation dominated the transcription pattern over the maternal mutation in these rare MATZYG embryos (Fig 8C and 8D). The MATMUT6 embryos displayed a highly variable pattern of TEs (Fig 8D).

### EHMT2 targets DNA methylation to suppress transcription from ERVKs over long distances and along multitudes of ‘passenger’ repeats

**Fig 9:**
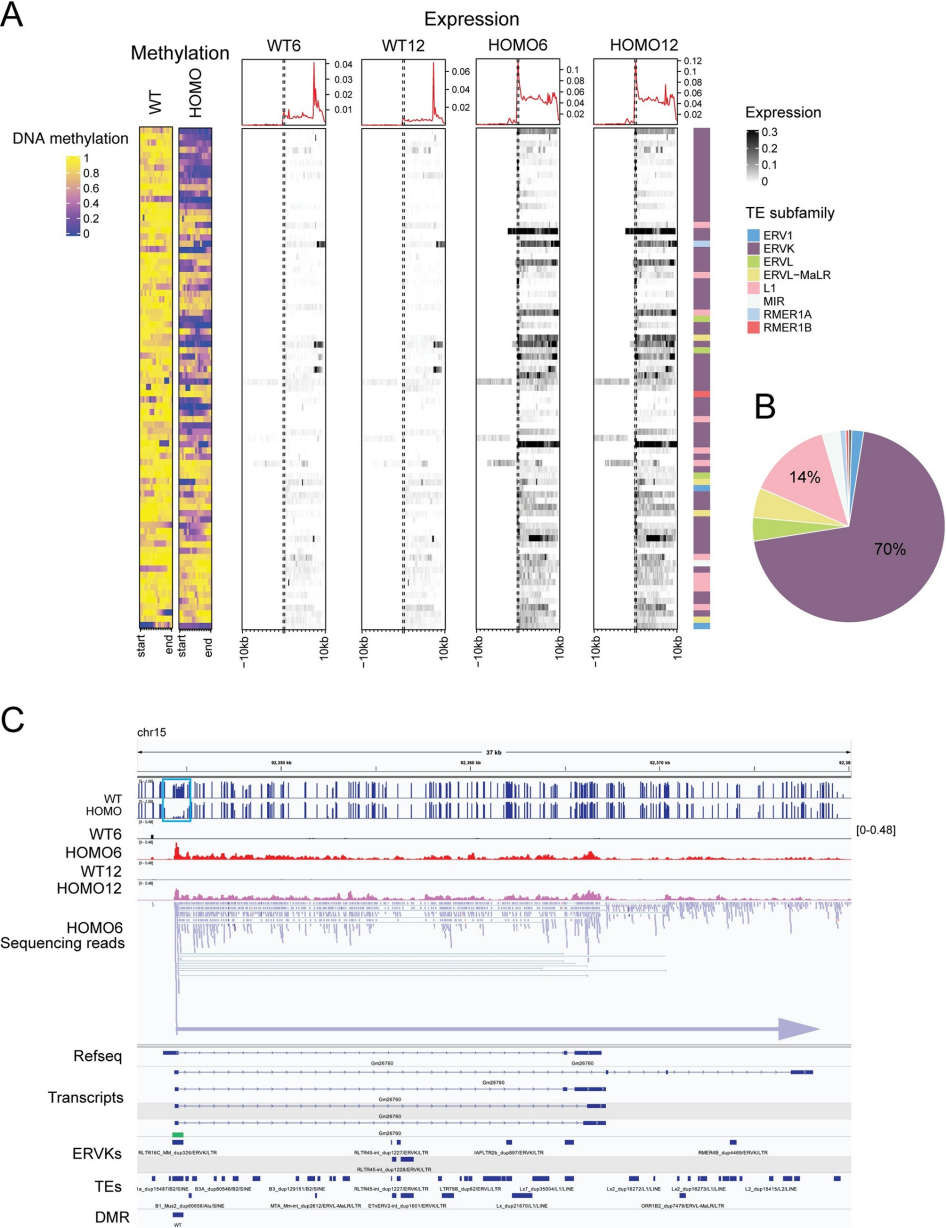
EHMT2 is required for DNA methylation targeting and suppression of transcription from transposable elements predominantly of the ERVK-class. (**A**) Heatmaps of differentially methylated TEs associated with differentially expressed long transcripts. DNA methylation is displayed at TE-DMRs in WT and HOMO embryos (to the left) and transcription is displayed along 10 kilobases before and after each TE in the embryos marked at the top. Normalized expression levels (logCPM) and the TE families are shown to the right. (**B**) Pie chart displays the proportion of DE repeat subfamilies associated with DNA methylation change using the color code as above. (**C**) Example for transcription initiation from an ERVK DMR in HOMO6 and HOMO12 embryos. IGV browser images of WGBS and RNAseq experiments are displayed in samples indicated to the left. The coverage is displayed for one WT6, HOMO6, WT12 and HOMO12 embryo, and the sequencing reads are displayed for one HOMO6 sample. Tracks of Refseq transcripts, putative transcripts, ERVKs, TEs, and DMRs are also included as marked to the left. A blue horizontal arrow indicates the long noncoding transcript in chr15 defined by sequencing reads (all blue) in the sense direction. It starts in an RLTR16C ERVK repeat (green rectangle), which is a DMR (turquoise rectangle), and it matches the putative transcript Gm26760. It encompasses multiple ‘passenger’ repeats, which also appear to be DE repeats, but which only display sequencing reads in the sense direction (blue) irrespective of their initial directionality.

When we sorted the misregulated repeats by chromosomal location, we noticed large clusters of many kinds of repeats following the same misexpression pattern, for example of being derepressed in HOMO embryos (S10 Table). We often found that a cluster of misexpressed TEs follows the expression pattern of one ‘driver’ TE, which displays a DMR between WT and HOMO embryos. The result of this analysis is displayed in Fig 9A. We show TE-DMRs in WT and HOMO embryos and transcription along 10 kilobases before and after each TE. The ERVK subfamily contributed 70% of the 145 ‘driver’ DMR-TE-s (Fig 9B). One particular TE from the ERVK family, called RLTR17 alone made up 22% of the DMR TEs. However, not all RLTR17, but only a small fraction (18/1739) were hypomethylated in HOMO embryos. Activation of transcription that initiated at these DMR TE-s spanned long distances, even over hundreds of kilobases. One example is shown in Fig 9C, where transcription that matches the putative non-coding RNA Gm26760 initiates from an RLTR17 ERVK DMR in HOMO6 and HOMO12 samples. The second example (S7 Fig) is transcription matching the putative transcript Gm13467 which initiates in another RLTR17 ERVK DMR (S7A–S7C Fig). The third example is transcription that matches the putative transcript 4933436I20Rik, initiating from an RLTR17 ERVK DMR in HOMO6 and HOMO12 embryos (S8A–S8C Fig). These three putative long transcripts run through numerous ‘passenger’ DE repeats of many kinds (S10 Table and Figs 9C, S7C and S8C). Interestingly, sequencing reads are only detectable in one direction along these long transcripts, in the + strand at Gm26760 and Gm13467, but only in the–DNA strand at 4933436I20Rik. Transcription, therefore, occurs along DE ‘passenger’ repeats but irrespective of the initial directionality of those ‘passenger’ repeats. These results collectively demonstrate that EHMT2 is an important regulator of TEs in the embryo and has high specificity to suppressing certain ERVKs in the embryo.

## Discussion

Understanding the effect of developmental delay is challenging. Even though we showed clear consequences of EHMT2 deficiency, it is very hard to distinguish primary from secondary effects at a broader level, even using stage or age-matched comparisons. One theoretical solution would be to compare different genetic mutants that show identical developmental delay to attempt to control for delay-dependent secondary effects. However, if such control was found for EHMT2, the primary effects could have been different as well. We approached this problem from another angle. We considered that the developmental progress and potential of the embryo depends on the dose of EHMT2 not only in the embryo but also in the gametes. We compared embryos delayed in development due to the different *Ehmt2* deficiencies, and we compared them when they reached the same developmental stage. We found that the different severity of developmental delay corresponds to the extent of transcriptomic changes in the embryo. By comparing zygotic and maternal effects and parental haploinsufficiency effects side-by-side we asked whether delayed development correlates with changes in the transcriptome that could predict the outcome of survival. We found that the transcriptome of HOMO embryos, which invariably die, is robustly distinguishable from WT embryos that reach the same somite-stage at an earlier time point. However, the transcriptomes are not altered in developmentally matched wild type MATHAPLO, PATHAPLO and WT (biparental haploinsufficient) embryos compared to CONTROL embryos. This negative result is a significant finding and suggests that these embryos develop slowly but otherwise normally after they have overcome an initial delay. Such cases likely occur during human pregnancies, when the fetus would have a slower and longer gestation despite being genetically normal. MATMUT embryos, however, that lacked EHMT2 in the oocyte, suffered lasting effects into the 6-somite stage. DEGs involved in erythrocyte and myeloid cell homeostasis were not properly suppressed and genes involved in mesoderm development, endoderm development, somitogenesis, and regionalization were not properly activated. In addition, higher variability was observed for genes in the categories of erythrocyte and myeloid cell homeostasis. We speculate that embryos, which have less transcriptional variation at the 6-somite stage, have a better chance of proceeding through development. The zygotic mutation-specific pattern dominated over the maternal mutation pattern in the rare MATZYG embryos. We speculate that had they not inherited the mutant allele from the father, they would likely belong to those MATMUT embryos that are lightly affected and develop to term. Having little effect from the maternal mutation may be required for a MATZYG embryo to develop to the 6-somite stage.

The exact timing of the blockage that initiates the delay in the different *Ehmt2* deficiencies will require further experiments. The 6.25 dpc and 8.5 dpc stages are already delayed in HOMO embryos [22,23] and the blastocyst stage may already be delayed in MATMUT embryos [2,14]. To understand transcriptional changes in mutant embryos that are delayed in development we applied two different strategies that considered the progress of embryo development. We focused on the effects of EHMT2 in the embryo at 8.5–9.5 dpc using somite matched embryos of different genotypes. A previous RNAseq study of *Ehmt2*^*−/−*^ embryos at 8.5 dpc [23] identified 253 DEGs with elevated and 181 DE genes with reduced expression in the *Ehmt2*^*−/−*^ mutant embryos. The authors focused on those DEGs that required EHMT2 for suppression. The large number of genes that appeared to require EHMT2 for activation in WT embryos remained unexplained in that study. Our study provides an explanation for this class of DEGs. They reflect the developmental changes from the 6- to 12-somite stage in WT embryos, highlighting the usefulness of our three-way comparison. Recently, a large set of mutant mouse lines were analyzed at 9.5 dpc by applying a three-way concept. Mutant embryos were contrasted against somite-staged wild type embryos from a universal baseline developmental dataset [38]. This method improved the detection of real DEGs that are in direct response to the mutation. It also excluded false positive hits that corresponded to normal development. However, it did not allow detecting the changes that occur specifically during the development of the wild type but not the mutant embryo. Our 4-way comparison uniquely identified developmental DEGs that require EHMT2 for their precise suppression or precise activation in normal development between the 6- and 12 somite stages. The 75 DEGs that need EHMT2 for their precise suppression include genes, such as *Nanog*, *Mesp1*, *Nodal*, *Hnf1b*, that are related to gastrulation, whereas the 191 DEGs that require EHMT2 for their precise activation include genes, such as *Erbb4*, *Neurog2*, *Wnt7b*, *Wnt8b*, *Myf5*, and *Fgf9*, relevant for organ growth, connective tissue development, striated muscle development, muscle differentiation, and cartilage development. These findings are consistent with a role of EHMT2 in regulating the transition between gastrulation and tissue/organ specification.

Our results showed DNA methylation-mediated suppression of germ cell-specific transcripts requires EHMT2 in the embryo, in agreement with an RRBS experiment [23]. Our comparative analysis revealed in addition that EHMT2-dependent DNA methylation is specific to DE genes in the “what it takes to be normal” class, but not in the “what it takes to develop” class. This is consistent with the finding that developmental changes were more often up-regulations, being released from a suppressing mechanism directly or indirectly imposed by EHMT2. DNA methylation at TEs does not globally require EHMT2 in the embryo as it does in ES cells [23]. Similarly, maternal EHMT2 has very limited effect on DNA methylation in the oocyte and in 2-cell embryos as assessed by WGBS [2]. Our WGBS results also found that EHMT2 has a limited effect on DNA methylation at TEs globally. However, EHMT2 specifically targets certain ERVKs for DNA methylation and suppression in the embryo. ERVKs are young repeat elements with high CpG content, and are under dual control of DNA methylation and H3K9 methylation [39]. Here we also revealed a unique feature of EHMT2-regulated ERVKs. Transcription could initiate in HOMO embryos from such hypomethylated repeats, and extend to several hundred kilobases, encompassing a multitude of additional, similarly misexpressed ‘passenger’ repeats, which do not display DNA hypomethylation. We show that 1) the ‘passenger’ TEs can be called DE irrespective of their directionality but the sequencing reads can only be detected in the sense direction of the long ERVK-driven transcript; and 2) the sequencing reads go beyond the region defined by the ‘passenger’ TEs. This means that the ‘passenger’ TEs may not have sufficient ability to express themselves and may not be by themselves responsive to EHMT2-mediated suppression.

The oocyte- or 2 cell-specific chimeric transcripts driven by class III retrotransposon LTRs [33–37] did not become derepressed in HOMO6 and HOMO12 embryos. This suggests that EHMT2 is not required for suppressing retrotransposon-driven oocyte/2-cell specific transcripts in the 6–12 somite stage embryos. Those LTRs may need a specific activation mechanism which is not available in the 6–12 somite stage embryos. By globally profiling transposable elements in the embryo using the 4-way comparisons we found that certain TE subclasses correlate with genotype, while others correlate with developmental stage. Contrary to DEGs, differentially expression of TEs most often correlated with the genotype. We found that ERV subfamilies, especially ERVK, ERVL-MaLR, ERV1, and ERVL, were the most affected, similar to what was found in 6.5 dpc *Ehmt2*^−/−^ zygotic mutant epiblasts [22]. These results cumulatively suggest that ERVs require EHMT2 for suppression in the 6.5–8.5 dpc mouse embryos, and H3K9me2 is likely involved.

Transcriptional noise in general is an important property of normal development at times when major decisions are made toward cell fates, such as in the process of early gastrulation [40]. Epigenetic modifiers regulate the transcriptional noise that underlies the inevitable phenotypic variation [41,42]. Maternally deposited TRIM28 is essential to carry out this role during preimplantation [43]. TET1 and TET3 curb transcriptional noise at the 8-cell and blastocyst stages [44]. We found it interesting that EHMT2 specifically reduces the noise of genes that switch developmental programs between gastrulation and organ specification. We found that EHMT2 is important in reducing the variation of developmental genes after gastrulation at the 6-somite stage. One group of genes was unusual, being developmental genes, such as *Nodal*, *Lefty1*, Lefty2, *Cdx1*, and *Foxh1*, which are suppressed by the 12 somite stages but showed variable suppression in HOMO6 embryos. These genes play a role in breaking the symmetry, which is an essential event in the turning of mouse embryos and in development of organ asymmetries [45]. Our results are consistent with the existence of a biological process that curbs the noise of developmental genes after gastrulation. Our results also identify EHMT2 as the regulator of this process. Further experiments will address this finding at the single cell level. Such a role of EHMT2 was also detected at transposable elements that switched off or on between 6-and 12-somite stages. It will be interesting to find out the timing when the transcriptional variation of such developmental genes is initiated during embryogenesis. The high variability of transcription in the majority of MATMUT embryos in the maternal allele suggests that these changes are initiated by EHMT2 depletion during oogenesis and are inherited to the next generation in the form of an epigenetic change. While the changes we describe depend on EHMT2, and its SET domain, it remains to be formally established whether these effects, including the effects on variation, correlate with H3K9 dimethylation at the target loci. In summary, our work now establishes EHMT2 as a regulator of transcriptional noise after gastrulation in a developmental context.

## Methods and models

### Ethics statement

All animal experiments were performed according to the National Institutes of Health Guide for the Care and Use of Laboratory animals, with Institutional Care and Use Committee-approved protocols at Van Andel Institute (VAI).

### Mice

An *Ehmt2* conditional knockout mouse line (*Ehmt2*^fl/fl^) was generated in our laboratory by gene targeting in 129S1/SvImJ ES cells [24]. The floxed SET domain was removed to generate the *Ehmt2*^−(129S1)^ allele by crossing the *Ehmt2*^fl/+^ male with 129S1/Sv-Hprt^tm1(CAGcre)Mann/J^ transgenic mouse female [46]. *Ehmt2*^fl/fl^ and *Ehmt2*^+/−(129S1)^ mice were maintained on 129S1 background. We backcrossed the *Ehmt2*^−(129S1)^ allele to the JF1/Ms (JF1) mouse strain [26] for more than ten generations and obtained the *Ehmt2*^−(JF1.N10)^ allele, where most of the genome has been replaced by JF1 chromosomes during meiotic recombination events except the *Ehmt2* locus and its close neighbors (in the approximate interval of chr 17:34647146–35241746), as confirmed in the allele-specific expression analysis in 12-somite embryos [25].

*Ehmt2*^−/−^ and WT embryos were obtained by natural mating of *Ehmt2*^+/−^ females with *Ehmt2*^+/−^ males. Having *Ehmt2*^+/−^ mice in both 129S1 and JF1 background allowed us to collect samples from reciprocally crossed F1 hybrids. Crossing *Ehmt2*^*+/*−(JF1.N10)^ females with *Ehmt2*^*+/*−(129S1)^ males yielded *Ehmt2*^−(JF1.N10)/−(129S1)^ zygotic mutant and *Ehmt2*^+(JF1)/+(129S1)^ wild type embryos. The reciprocal cross resulted in *Ehmt2*^−(129S1)/−(JF1.N10)^ mutant and *Ehmt2*^+(129S1)/+(JF1)^ wild type (biparental haploinsufficient) embryos. Control *Ehmt2*^+(129S1)/+(JF1)^ embryos were obtained by crossing wild type 129S1 and JF1 male mice.

*Ehmt2*^+/− (JF1.N10)^ females were crossed with wild-type 129S1 males to generate maternal haploinsufficient wild type *Ehmt2*^+ (JF1)/+(129S1)^ embryos. *Ehmt2*^+/−(129S1)^ males were crossed with wild-type JF1 females to generate paternal haploinsufficient wild type *Ehmt2*^+(JF1)/+ (129S1)^ embryos. Control *Ehmt2*^+(JF1)/+ (129S1)^ embryos were obtained by crossing wild type JF1 female and 129S1 male mice.

*Ehmt2*^fl/fl^; Zp3-cre^Tg/0^ females were crossed with wild type JF1 males to generate *Ehmt2*^mat−/+JF1^ maternal mutant embryos. Their control came from crossing *Ehmt2*^fl/fl^ females with wild type JF1 males. *Ehmt2*^fl/fl^; Zp3-cre^Tg/0^ females were crossed with *Ehmt2*^*+/*−(JF1.N10)^ males to generate *Ehmt2*^mat−/zyg−(JF1)^ maternal-zygotic mutant embryos.

Embryos were dissected at 8.5 dpc and 9.5 dpc, and were genotyped for *Ehmt2* mutation status and for sex by PCR using their allantois as described [25].

### RNA isolation and sequencing

RNA was isolated from individual embryo samples using RNA-Bee (Tel-Test) extraction followed by isopropanol precipitation. Genomic DNA was removed with the DNA-free Kit (Ambion). Total RNA-seq libraries were prepared from 500 ng total RNA with the KAPA Stranded RNA-Seq Kit with RiboErase (Kapa Biosystems, MA), and were sequenced in the Illumina NextSeq 500 platform with paired-end 75 bp read length. We obtained at least 40 M reads for each embryo sample, and 20 M reads were uniquely aligned.

### RNAseq data analysis

Paired-end reads were aligned to the mm10 genome, using STAR (v 2.6.0c) [47] with parameters—outFilterType BySJout—outFilterMultimapNmax 20—alignSJoverhangMin 8—alignSJDBoverhangMin 1—alignIntronMax 1000000—alignMatesGapMax 1000000—outFilterMismatchNmax 999—twopassMode Basic—chimSegmentMin 20—alignIntronMin 20. Using the ensembl gtf file (version 82), read counts per transcript was generated using featureCounts (-P -s 1—primary -Q 20 –ignoreDup). Using the Ensembl gene annotation file (v82), counts per gene annotation were generated using featureCounts. Genes with at least CPM > 1 in at least 2 of the samples were retained for analysis.

Quality control: RNA-Seq reads were trimmed to remove low-quality and adapter bases using the TrimGalore v0.6.0 (https://github.com/FelixKrueger/TrimGalore) wrapper for CutAdapt v2.10 [48]. Alignment and read counting was done using STAR v2.7.8a [47] against the mm10 reference and GENCODE vM24 annotations [49]. The heatmaps were generated using pheatmap v1.0.12 [50] with TMM-normalized log2 CPM values calculated using edgeR v3.34.0 [51]. Sequence quality of the samples was determined after quality-trimming, parsed from FastQC v0.11.9 (https://www.bioinformatics.babraham.ac.uk/projects/fastqc/) reports using the R package, ngsReports v1.4.2 [52]. In addition, the STAR v2.7.8a [47] alignment rates were also parsed against mm10 from the MultiQC v1.8 [53] output.

Differential expression was performed using edgeR [51] and differentially expressed genes were identified using the cutoff values of FDR < 0.05 and the absolute value of the logFC > 1.2. Venn diagrams were generated using http://bioinformatics.psb.ugent.be/webtools/Venn/. Enriched gene ontology terms were determined using the “clusterProfiler” package [54] in R. A term was defined as enriched applying the adj.pvalue < 0.05.

Principle component analysis was performed on DEGs using the prcomp function in R. The first two PC’s were plotted using the ggfortify package in R.

Group-wise average variance and coefficients of variation were calculated from normalized counts produced by the variance stabilizing transformation (VST) approach implemented in DESeq2 v1.32.0 [55]. A differential variability analysis was performed using MDSeq [31] to determine differentially variable genes within each comparison (WT12 vs WT6, HOMO12 vs HOMO6, WT6 vs HOMO6, and WT12 vs HOMO12). Differential variation was defined as a FDR.dispersion < 0.05 and a logFC > abs(1.5). Similarly, a gene was determined as differentially variable between the MATMUT and MATWT using MDSeq, and cutoff value of FDR dispersion < 0.05. The heatmap was generated using the Complexheatmap [56] package in R.

GO enrichment was performed on the differentially variable genes using the clusterProfiler [54] package. The most significant terms were plotted using the ggplot2 package in R.

To identify DE TE families, the TEtranscripts workflow was used [57]. Trimmed reads were aligned to mm10 using STAR v2.7.8a with GENCODE vM24 annotations and the parameters, “—winAnchorMultimapNmax 200” and “—outFilterMultimapNmax 100”. Gene and TE counts were obtained using ‘TEcount’ from TEtranscripts v2.2.1 [58] with a TE GTF curated by the TEtranscripts authors. Gene and TE counts were tested for DE using DESeq2 as described above.

Uniquely mapped reads were extracted (samtools view -f 255), featurecounts was used to count reads per repeatmasker [59] annotation, and differential repeat analysis was performed using edgeR.

### Whole genome bisulfite sequencing (WGBS)

WGBS analysis was used to map DNA methylation at 9.5 dpc. One sample contains DNA from two *Ehmt2* homozygous mutant embryos (one male and one female combined), and the control sample contains DNA from a wild type female embryo. In order to generate the whole genome libraries with bisulfite converted DNA, embryo DNA was sonicated to approx. 150 bp DNA fragments. Further, DNA was end repaired by using End-It- DNA End-Repair Kit (Epicentre) and linker ligated with T4 ligase (NEB). The linked ligated DNA was bisulfite converted using EZ DNA Methylation-Gold Kit according to manufacturer’s instructions (Zymo Research) and amplified with Pfu Turbo polymerase (Agilent). Sequencing was performed in the Illumina HiSeq 2500 platform at the Integrative Genomic Core at the City of Hope Cancer Center.

Pair-end reads were aligned to the mm10 genome using Biscuit and duplicates were marked using Picard’s MarkDuplicates. DNA methylation and genetic information were extracted using biscuit’s pileup and CpG beta values were extracted using vcf2bed. Significant DMR at the TSS +/- 1000 bp were determined using metilene with options–mode 2. A custom R script was used to determine genes that overlap with significant TSS +/-1000 bp locations. Differently methylated regions were de novo identified using metilene v0.2–8 with parameters–mode 2. A region was identified as differentially methylated when it contained at least 12 CpG-s and reached the Benjamini-Hochberg adjusted p-value of *P*< 0.05. Significant regions in the mm10 genome were annotated using Homers annotatePeaks.pl.

### Statistics

To detect variance in development we applied the Friedman non-parametric test. This was necessary because the sample sizes were small and normality checks showed that the assumption of normality had not been met.

## Supporting information

S1 Fig Read depth and quality control of the RNAseq results. FastQC analysis is shown. (**A**) Total reads, unique and duplicated reads are plotted for each embryo sample in the R1 direction. (**B**) Mean sequence quality is plotted for each embryo sample in the R1 direction. (**C**) Sequence length distribution is plotted for each embryo sample in the R1 direction. (**D**) Total reads, unique and duplicated reads are plotted for each embryo sample in the R2 direction. (**E**) Mean sequence quality is plotted for each embryo sample in the R2 direction. (**F**) Sequence length distribution is plotted for each embryo sample in the R4 direction.
(TIF)

S2 Fig Sequencing homogeneity of the RNAseq results. (**A**) Heatmap displays the results of an unsupervised cluster analysis that calculates Eucledian distance between samples based on all transcripts. Sample types are color-coded and normalized log2 cpm values are color-coded as shown to the right. Sample IDs are given at the bottom. Note the uniformity of the dataset. (**A**) Heatmap displays the results of an unsupervised cluster analysis that calculates Eucledian distance between samples based on the top 1000 variable transcripts. Note the split of the HOMO and MATZYG samples from the remaining samples. Also note the high variability of the HOMO6, MATZYG6, and MATMUT samples.
(TIF)

S3 Fig Stratifying DMRs. A WGBS experiment was performed comparing 9.5 dpc WT and HOMO embryos. DMRs were called and stratified into genomic elements as shown by colors. (**A**) DMRs that require EHMT2 for DNA hypermethylation. (**B**) DMRs that require EHMT2 for DNA hypomethylation.
(TIF)

S4 Fig Identifying what it takes to be normal and what it takes to develop. Heatmaps display the DE genes from the respective segments of the 4-way Venn diagrams depicted in Fig 3D and 3E. Arrows indicated the direction of changes with the number of DE genes in those sections. Below the heatmaps IGV browser examples are shown for the DEGs that drive the GO term in those specific Venn segments. Samples are labeled at the top with the color code shown to the right. Expression values (logCPM) are shown according to the scale to the right. (**A-D**) What it takes to be normal. Changes occur in response to EHMT2 at both the 6-somite and 12-somite stages. (**E**-**H**) What it takes to develop. Developmental DEGs downregulated (**E**) or upregulated (**F**) between the 6-and 12-somite stages regardless of EHMT2. Other details are as explained in Fig 3.
(TIF)

S5 Fig High variability of transcription is determined by the maternal allele in MATMUT embryos. IGV browser images of the maternal (MAT) and paternal (PAT) alleles are shown of each representative transcript that exhibit high variability in MATMUT embryos in four replicate samples per each genotype. The transcription profile of the maternal (MAT) and paternal (PAT) alleles are shown of each transcript in four replicate samples per each genotype. (**A**), Both alleles are derepressed in MATMUT embryos, and the maternal allele is more derepressed than the paternal allele (**B**) The control gene *Gapdh* shows no variation in MATMUT embryos. (**C**) The maternal allele is derepressed in MATMUT embryos while the paternal allele is unchanged. (**D**) The maternal allele of imprinted genes is derepressed in MATMUT embryos while the paternal allele remains silent.
(TIF)

S6 Fig EHMT2-regulated repeat elements identified by the 4-way comparison. Uniquely mapped DE repeats were identified in the four-way comparison. (**A-F**) Heatmaps display the transcription level (logCPM) of those uniquely mapped repeats that are differentially expressed in the comparisons indicated at the top. Samples are shown by the color code to the right. The classification of each DE repeat is indicated in the pie charts underneath the respective heatmaps.
(TIF)

S7 Fig Transcription initiates from a hypomethylatd ERVK in HOMO embryos. (**A**) Heatmap of differentially expressed TEs (as marked to the left) is depicted in the samples (as marked at the top) along a segment of chromosome 2. DNA methylation is displayed at TE-DMRs in WT and HOMO embryos (to the right). A long noncoding transcript is predicted (gray shading) by the synchronously misregulated ‘passenger’ repeats. It starts in an RLTR17 ERVK repeat (green rectangle), which is a DMR (turquoise rectangle), and it encompasses multiple ‘passenger’ repeats, which also appear to be DE repeats, irrespective of their initial directionality (strand). Blue vertical arrow indicates the long noncoding transcript that is defined by sequencing reads in the sense direction, as shown below. (**B**) Transcription initiates from an ERVK DMR in HOMO6 and HOMO12 embryos. One example is shown. IGV browser images of WGBS and RNAseq experiments are displayed in samples indicated to the left. The sequencing reads are displayed for the HOMO6 sample. Tracks of Refseq transcripts, putative transcripts, ERVKs, TEs, and DMRs are also included as marked to the left. Blue horizontal arrow indicates the long noncoding transcript that is defined by sequencing reads in the sense direction. It starts in an RLTR17 ERVK repeat (green rectangle), which is a DMR (turquoise rectangle), and it matches the putative transcript Gm13467. It encompasses multiple ‘passenger’ repeats, which also appear to be DE repeats, but which only display sequencing reads in the sense direction (blue) irrespective of their initial directionality. Part of the image is marked with black rectangle to be shown in more detail to the right. (**C**) Enlarged detail shows the initiation of the long noncoding transcript.
(TIF)

S8 Fig Transcription initiates from a hypomethylatd ERVK in HOMO embryos in the minus DNA strand. (**A**) Heatmap of differentially expressed TEs (as marked to the left) is depicted in the samples (as marked at the top) along a segment of chromosome 1. DNA methylation is displayed at TE-DMRs in WT and HOMO embryos (to the right). A long noncoding transcript is predicted (gray shading) by the synchronously misregulated ‘passenger’ repeats. It starts in an RLTR17 ERVK repeat (green rectangle), which is a DMR (turquoise rectangle), and it encompasses multiple ‘passenger’ repeats, which also appear to be DE repeats, irrespective of their initial directionality (strand). Pink vertical arrow indicates the long noncoding transcript that is defined by sequencing reads in the antisense direction, as shown below. (**B**) Transcription initiates from an ERVK DMR in HOMO6 and HOMO12 embryos. One example is shown. IGV browser images of WGBS and RNAseq experiments are displayed in samples indicated to the left. The sequencing reads are displayed for the HOMO6 sample. Tracks of Refseq transcripts, putative transcripts, ERVKs, TEs, and DMRs are also included as marked to the left. Blue horizontal arrow indicates the long noncoding transcript that is defined by sequencing reads in the sense direction. It starts in an RLTR17 ERVK repeat (green rectangle), which is a DMR (turquoise rectangle), and it matches the putative transcript 4933436I20Rik. It encompasses multiple ‘passenger’ repeats, which also appear to be DE repeats, but which only display sequencing reads in the antisense direction irrespective of their initial directionality. Part of the image is marked with black rectangle to be shown in more detail below. (**C**) Enlarged detail shows the initiation of the long noncoding transcript.
(TIF)

S1 Table Surviving pups with *Ehmt2* maternal mutation. (XLSX)

S2 Table Summary of samples. (XLSX)

S3 Table Differentially expressed genes in the three-way comparison. (XLSX)

S4 Table Genome-wide DMRs identified in WGBS. (XLSX)

S5 Table DEGs identified in the four-way comparison and in MATMUT, MATHAPLO and PATHAPLO embryos. (XLSX)

S6 Table Gene ontology analysis of DEGs identified in the four-way comparison. (XLSX)

S7 Table Gene ontology analysis of *Ehmt2* maternal mutant embryos by DEGs and dispersion. (XLSX)

S8 Table Genes displaying significant dispersion in *Ehmt2* maternal mutant embryos. (XLSX)

S9 Table The effect of *Ehmt2* mutation on repeat families. (XLSX)

S10 Table The effect of EHMT2 on the expression and DNA methylation of uniquely mapped repeats. (XLSX)

## References

[1] Ancelin K, Syx L, Borensztein M, Ranisavljevic N, Vassilev I, Briseno-Roa L, et al. Maternal LSD1/KDM1A is an essential regulator of chromatin and transcription landscapes during zygotic genome activation. eLife. 2016;5. doi: 10.7554/eLife.08851 ; PubMed Central PMCID: PMC4829419. 26836306 PMC4829419

[2] Au Yeung WK, Brind’Amour J, Hatano Y, Yamagata K, Feil R, Lorincz MC, et al. Histone H3K9 Methyltransferase G9a in Oocytes Is Essential for Preimplantation Development but Dispensable for CG Methylation Protection. Cell Rep. 2019;27(1):282–93 e4. doi: 10.1016/j.celrep.2019.03.002 . 30943408

[3] Bai L, Yang L, Zhao C, Song L, Liu X, Bai C, et al. Histone Demethylase UTX is an Essential Factor for Zygotic Genome Activation and Regulates Zscan4 Expression in Mouse Embryos. International journal of biological sciences. 2019;15(11):2363–72. doi: 10.7150/ijbs.34635 ; PubMed Central PMCID: PMC6775313. 31595154 PMC6775313

[4] Brici D, Zhang Q, Reinhardt S, Dahl A, Hartmann H, Schmidt K, et al. Setd1b, encoding a histone 3 lysine 4 methyltransferase, is a maternal effect gene required for the oogenic gene expression program. Development. 2017;144(14):2606–17. doi: 10.1242/dev.143347 . 28619824

[5] Ciccone DN, Su H, Hevi S, Gay F, Lei H, Bajko J, et al. KDM1B is a histone H3K4 demethylase required to establish maternal genomic imprints. Nature. 2009;461(7262):415–8. Epub 2009/09/04. nature08315 [pii] doi: 10.1038/nature08315 . 19727073

[6] Dahl JA, Jung I, Aanes H, Greggains GD, Manaf A, Lerdrup M, et al. Broad histone H3K4me3 domains in mouse oocytes modulate maternal-to-zygotic transition. Nature. 2016;537(7621):548–52. doi: 10.1038/nature19360 . 27626377 PMC6283663

[7] Eymery A, Liu Z, Ozonov EA, Stadler MB, Peters AH. The methyltransferase Setdb1 is essential for meiosis and mitosis in mouse oocytes and early embryos. Development. 2016;143(15):2767–79. doi: 10.1242/dev.132746 . 27317807

[8] Inoue A, Chen Z, Yin Q, Zhang Y. Maternal Eed knockout causes loss of H3K27me3 imprinting and random X inactivation in the extraembryonic cells. Genes Dev. 2018;32(23–24):1525–36. doi: 10.1101/gad.318675.118 ; PubMed Central PMCID: PMC6295166. 30463900 PMC6295166

[9] Jimenez R, Melo EO, Davydenko O, Ma J, Mainigi M, Franke V, et al. Maternal SIN3A regulates reprogramming of gene expression during mouse preimplantation development. Biology of reproduction. 2015;93(4):89. doi: 10.1095/biolreprod.115.133504 ; PubMed Central PMCID: PMC4711907. 26353893 PMC4711907

[10] Kim J, Zhao H, Dan J, Kim S, Hardikar S, Hollowell D, et al. Maternal Setdb1 Is Required for Meiotic Progression and Preimplantation Development in Mouse. PLoS Genet. 2016;12(4):e1005970. doi: 10.1371/journal.pgen.1005970 ; PubMed Central PMCID: PMC4829257. 27070551 PMC4829257

[11] Sankar A, Lerdrup M, Manaf A, Johansen JV, Gonzalez JM, Borup R, et al. KDM4A regulates the maternal-to-zygotic transition by protecting broad H3K4me3 domains from H3K9me3 invasion in oocytes. Nat Cell Biol. 2020;22(4):380–8. doi: 10.1038/s41556-020-0494-z . 32231309 PMC7212036

[12] Wasson JA, Simon AK, Myrick DA, Wolf G, Driscoll S, Pfaff SL, et al. Maternally provided LSD1/KDM1A enables the maternal-to-zygotic transition and prevents defects that manifest postnatally. eLife. 2016;5. doi: 10.7554/eLife.08848 ; PubMed Central PMCID: PMC4829428. 26814574 PMC4829428

[13] Yang L, Song LS, Liu XF, Xia Q, Bai LG, Gao L, et al. The Maternal Effect Genes UTX and JMJD3 Play Contrasting Roles in Mus musculus Preimplantation Embryo Development. Scientific reports. 2016;6:26711. doi: 10.1038/srep26711 ; PubMed Central PMCID: PMC4935995. 27384759 PMC4935995

[14] Zylicz JJ, Borensztein M, Wong FC, Huang Y, Lee C, Dietmann S, et al. G9a regulates temporal preimplantation developmental program and lineage segregation in blastocyst. eLife. 2018;7. doi: 10.7554/eLife.33361 ; PubMed Central PMCID: PMC5959720. 29745895 PMC5959720

[15] Liao J, Szabo PE. Maternal DOT1L is dispensable for mouse development. Scientific reports. 2020;10(1):20636. Epub 2020/11/28. doi: 10.1038/s41598-020-77545-6 ; PubMed Central PMCID: PMC7691351. 33244015 PMC7691351

[16] Dodge JE, Kang YK, Beppu H, Lei H, Li E. Histone H3-K9 methyltransferase ESET is essential for early development. Mol Cell Biol. 2004;24(6):2478–86. doi: 10.1128/MCB.24.6.2478-2486.2004 . 14993285 PMC355869

[17] Jones B, Su H, Bhat A, Lei H, Bajko J, Hevi S, et al. The histone H3K79 methyltransferase Dot1L is essential for mammalian development and heterochromatin structure. PLoS Genet. 2008;4(9):e1000190. doi: 10.1371/journal.pgen.1000190 ; PubMed Central PMCID: PMC2527135 Biomedical Research. 18787701 PMC2527135

[18] Tachibana M, Ueda J, Fukuda M, Takeda N, Ohta T, Iwanari H, et al. Histone methyltransferases G9a and GLP form heteromeric complexes and are both crucial for methylation of euchromatin at H3-K9. Genes Dev. 2005;19(7):815–26. doi: 10.1101/gad.1284005 . 15774718 PMC1074319

[19] Daxinger L, Oey H, Isbel L, Whitelaw NC, Youngson NA, Spurling A, et al. Hypomethylation of ERVs in the sperm of mice haploinsufficient for the histone methyltransferase Setdb1 correlates with a paternal effect on phenotype. Scientific reports. 2016;6:25004. doi: 10.1038/srep25004 ; PubMed Central PMCID: PMC4845014. 27112447 PMC4845014

[20] Siklenka K, Erkek S, Godmann M, Lambrot R, McGraw S, Lafleur C, et al. Disruption of histone methylation in developing sperm impairs offspring health transgenerationally. Science. 2015;350(6261):aab2006. doi: 10.1126/science.aab2006 . 26449473

[21] Tachibana M, Sugimoto K, Nozaki M, Ueda J, Ohta T, Ohki M, et al. G9a histone methyltransferase plays a dominant role in euchromatic histone H3 lysine 9 methylation and is essential for early embryogenesis. Genes Dev. 2002;16(14):1779–91. doi: 10.1101/gad.989402 . 12130538 PMC186403

[22] Zylicz JJ, Dietmann S, Gunesdogan U, Hackett JA, Cougot D, Lee C, et al. Chromatin dynamics and the role of G9a in gene regulation and enhancer silencing during early mouse development. eLife. 2015;4. doi: 10.7554/eLife.09571 ; PubMed Central PMCID: PMC4729692. 26551560 PMC4729692

[23] Auclair G, Borgel J, Sanz LA, Vallet J, Guibert S, Dumas M, et al. EHMT2 directs DNA methylation for efficient gene silencing in mouse embryos. Genome Res. 2016;26(2):192–202. doi: 10.1101/gr.198291.115 ; PubMed Central PMCID: PMC4728372. 26576615 PMC4728372

[24] Zeng TB, Han L, Pierce N, Pfeifer GP, Szabó PE. EHMT2 and SETDB1 protect the maternal pronucleus from 5mC oxidation. Proc Natl Acad Sci U S A. 2019;116(22):10834–41. Epub 2019/05/16. doi: 10.1073/pnas.1819946116 ; PubMed Central PMCID: PMC6561192. 31088968 PMC6561192

[25] Zeng TB, Pierce N, Liao J, Szabo PE. H3K9 methyltransferase EHMT2/G9a controls ERVK-driven noncanonical imprinted genes. Epigenomics. 2021;13(16):1299–314. Epub 2021/09/15. doi: 10.2217/epi-2021-0168 . 34519223

[26] Koide T, Moriwaki K, Uchida K, Mita A, Sagai T, Yonekawa H, et al. A new inbred strain JF1 established from Japanese fancy mouse carrying the classic piebald allele. Mamm Genome. 1998;9(1):15–9. Epub 1998/01/22. doi: 10.1007/s003359900672 . 9434939

[27] Tran DA, Bai AY, Singh P, Wu X, Szabó PE. Characterization of the imprinting signature of mouse embryo fibroblasts by RNA deep sequencing. Nucleic Acids Res. 2014;42(3):1772–83. doi: 10.1093/nar/gkt1042 ; PubMed Central PMCID: PMC3919614. 24217910 PMC3919614

[28] Leung DC, Dong KB, Maksakova IA, Goyal P, Appanah R, Lee S, et al. Lysine methyltransferase G9a is required for de novo DNA methylation and the establishment, but not the maintenance, of proviral silencing. Proc Natl Acad Sci U S A. 2011;108(14):5718–23. doi: 10.1073/pnas.1014660108 ; PubMed Central PMCID: PMC3078371. 21427230 PMC3078371

[29] Tachibana M, Matsumura Y, Fukuda M, Kimura H, Shinkai Y. G9a/GLP complexes independently mediate H3K9 and DNA methylation to silence transcription. EMBO J. 2008;27(20):2681–90. doi: 10.1038/emboj.2008.192 ; PubMed Central PMCID: PMC2572175. 18818694 PMC2572175

[30] Dong KB, Maksakova IA, Mohn F, Leung D, Appanah R, Lee S, et al. DNA methylation in ES cells requires the lysine methyltransferase G9a but not its catalytic activity. EMBO J. 2008;27(20):2691–701. doi: 10.1038/emboj.2008.193 ; PubMed Central PMCID: PMC2572176. 18818693 PMC2572176

[31] Ran D, Daye ZJ. Gene expression variability and the analysis of large-scale RNA-seq studies with the MDSeq. Nucleic Acids Res. 2017;45(13):e127. doi: 10.1093/nar/gkx456 ; PubMed Central PMCID: PMC5737414. 28535263 PMC5737414

[32] Di Giacomo M, Comazzetto S, Sampath SC, Sampath SC, O’Carroll D. G9a co-suppresses LINE1 elements in spermatogonia. Epigenetics & chromatin. 2014;7:24. doi: 10.1186/1756-8935-7-24 ; PubMed Central PMCID: PMC4177377. 25276231 PMC4177377

[33] Maksakova IA, Thompson PJ, Goyal P, Jones SJ, Singh PB, Karimi MM, et al. Distinct roles of KAP1, HP1 and G9a/GLP in silencing of the two-cell-specific retrotransposon MERVL in mouse ES cells. Epigenetics & chromatin. 2013;6(1):15. doi: 10.1186/1756-8935-6-15 ; PubMed Central PMCID: PMC3682905. 23735015 PMC3682905

[34] Macfarlan TS, Gifford WD, Driscoll S, Lettieri K, Rowe HM, Bonanomi D, et al. Embryonic stem cell potency fluctuates with endogenous retrovirus activity. Nature. 2012;487(7405):57–63. doi: 10.1038/nature11244 ; PubMed Central PMCID: PMC3395470. 22722858 PMC3395470

[35] Peaston AE, Evsikov AV, Graber JH, de Vries WN, Holbrook AE, Solter D, et al. Retrotransposons regulate host genes in mouse oocytes and preimplantation embryos. Dev Cell. 2004;7(4):597–606. doi: 10.1016/j.devcel.2004.09.004 . 15469847

[36] Veselovska L, Smallwood SA, Saadeh H, Stewart KR, Krueger F, Maupetit-Mehouas S, et al. Deep sequencing and de novo assembly of the mouse oocyte transcriptome define the contribution of transcription to the DNA methylation landscape. Genome biology. 2015;16:209. doi: 10.1186/s13059-015-0769-z ; PubMed Central PMCID: PMC4582738. 26408185 PMC4582738

[37] Macfarlan TS, Gifford WD, Agarwal S, Driscoll S, Lettieri K, Wang J, et al. Endogenous retroviruses and neighboring genes are coordinately repressed by LSD1/KDM1A. Genes Dev. 2011;25(6):594–607. Epub 2011/03/02. doi: 10.1101/gad.2008511 ; PubMed Central PMCID: PMC3059833. 21357675 PMC3059833

[38] Collins JE, White RJ, Staudt N, Sealy IM, Packham I, Wali N, et al. Common and distinct transcriptional signatures of mammalian embryonic lethality. Nature communications. 2019;10(1):2792. doi: 10.1038/s41467-019-10642-x ; PubMed Central PMCID: PMC6594971. 31243271 PMC6594971

[39] Ohtani H, Liu M, Zhou W, Liang G, Jones PA. Switching roles for DNA and histone methylation depend on evolutionary ages of human endogenous retroviruses. Genome Res. 2018;28(8):1147–57. doi: 10.1101/gr.234229.118 ; PubMed Central PMCID: PMC6071641. 29970451 PMC6071641

[40] Mohammed H, Hernando-Herraez I, Savino A, Scialdone A, Macaulay I, Mulas C, et al. Single-Cell Landscape of Transcriptional Heterogeneity and Cell Fate Decisions during Mouse Early Gastrulation. Cell Rep. 2017;20(5):1215–28. doi: 10.1016/j.celrep.2017.07.009 ; PubMed Central PMCID: PMC5554778. 28768204 PMC5554778

[41] Blewitt ME, Chong S, Whitelaw E. How the mouse got its spots. Trends Genet. 2004;20(11):550–4. Epub 2004/10/12. doi: 10.1016/j.tig.2004.08.011 . 15475114

[42] Whitelaw NC, Chong S, Whitelaw E. Tuning in to noise: epigenetics and intangible variation. Dev Cell. 2010;19(5):649–50. doi: 10.1016/j.devcel.2010.11.001 . 21074715

[43] Messerschmidt DM, de Vries W, Ito M, Solter D, Ferguson-Smith A, Knowles BB. Trim28 is required for epigenetic stability during mouse oocyte to embryo transition. Science. 2012;335(6075):1499–502. Epub 2012/03/24. 335/6075/1499 [pii] doi: 10.1126/science.1216154 . 22442485

[44] Kang J, Lienhard M, Pastor WA, Chawla A, Novotny M, Tsagaratou A, et al. Simultaneous deletion of the methylcytosine oxidases Tet1 and Tet3 increases transcriptome variability in early embryogenesis. Proc Natl Acad Sci U S A. 2015;112(31):E4236–45. Epub 2015/07/23. doi: 10.1073/pnas.1510510112 ; PubMed Central PMCID: PMC4534209. 26199412 PMC4534209

[45] Robertson EJ. Dose-dependent Nodal/Smad signals pattern the early mouse embryo. Seminars in cell & developmental biology. 2014;32:73–9. doi: 10.1016/j.semcdb.2014.03.028 . 24704361

[46] Tang SH, Silva FJ, Tsark WM, Mann JR. A Cre/loxP-deleter transgenic line in mouse strain 129S1/SvImJ. Genesis. 2002;32(3):199–202. doi: 10.1002/gene.10030 . 11892008

[47] Dobin A, Davis CA, Schlesinger F, Drenkow J, Zaleski C, Jha S, et al. STAR: ultrafast universal RNA-seq aligner. Bioinformatics. 2013;29(1):15–21. Epub 2012/10/30. doi: 10.1093/bioinformatics/bts635 ; PubMed Central PMCID: PMC3530905. 23104886 PMC3530905

[48] Martin M. Cutadapt removes adapter sequences from high-throughput sequencing reads. EMBnet journal. 2011;17(1):10–2.

[49] Frankish A, Diekhans M, Ferreira AM, Johnson R, Jungreis I, Loveland J, et al. GENCODE reference annotation for the human and mouse genomes. Nucleic Acids Res. 2019;47(D1):D766–D73. Epub 2018/10/26. doi: 10.1093/nar/gky955 ; PubMed Central PMCID: PMC6323946. 30357393 PMC6323946

[50] Kolde R. Package ‘pheatmap.’. R Package. 2015.

[51] Robinson MD, McCarthy DJ, Smyth GK. edgeR: a Bioconductor package for differential expression analysis of digital gene expression data. Bioinformatics. 2010;26(1):139–40. doi: 10.1093/bioinformatics/btp616 ; PubMed Central PMCID: PMC2796818. 19910308 PMC2796818

[52] Ward CM, To TH, Pederson SM. ngsReports: a Bioconductor package for managing FastQC reports and other NGS related log files. Bioinformatics. 2020;36(8):2587–8. Epub 2019/12/17. doi: 10.1093/bioinformatics/btz937 . 31841127

[53] Ewels P, Magnusson M, Lundin S, Kaller M. MultiQC: summarize analysis results for multiple tools and samples in a single report. Bioinformatics. 2016;32(19):3047–8. Epub 2016/06/18. doi: 10.1093/bioinformatics/btw354 ; PubMed Central PMCID: PMC5039924. 27312411 PMC5039924

[54] Yu G, Wang LG, Han Y, He QY. clusterProfiler: an R package for comparing biological themes among gene clusters. Omics: a journal of integrative biology. 2012;16(5):284–7. doi: 10.1089/omi.2011.0118 ; PubMed Central PMCID: PMC3339379. 22455463 PMC3339379

[55] Love MI, Huber W, Anders S. Moderated estimation of fold change and dispersion for RNA-seq data with DESeq2. Genome biology. 2014;15(12):550. Epub 2014/12/18. doi: 10.1186/s13059-014-0550-8 ; PubMed Central PMCID: PMC4302049. 25516281 PMC4302049

[56] Gu Z, Eils R, Schlesner M. Complex heatmaps reveal patterns and correlations in multidimensional genomic data. Bioinformatics. 2016;32(18):2847–9. doi: 10.1093/bioinformatics/btw313 . 27207943

[57] Jin Y, Hammell M. Analysis of RNA-Seq Data Using TEtranscripts. Transcriptome Data Analysis: Methods and Protocols Springer Y Wang & M Sun (Eds). 2018:153–67. doi: 10.1007/978-1-4939-7710-9_11 29508296

[58] Jin Y, Tam OH, Paniagua E, Hammell M. TEtranscripts: a package for including transposable elements in differential expression analysis of RNA-seq datasets. Bioinformatics. 2015;31(22):3593–9. Epub 2015/07/25. doi: 10.1093/bioinformatics/btv422 ; PubMed Central PMCID: PMC4757950. 26206304 PMC4757950

[59] Smit AFA, Hubley R, Green P. http:// www.repeatmasker.org. RepeatMasker Open-40 2013–2015.

