# Supplementary figures and images for: EHMT2 suppresses the variation of transcriptional switches in the mouse embryo
Source: PLoS Genet. 2021 Nov 18;17(11):e1009908. doi: 10.1371/journal.pgen.1009908 (PMC8601470; doi:10.1371/journal.pgen.1009908)

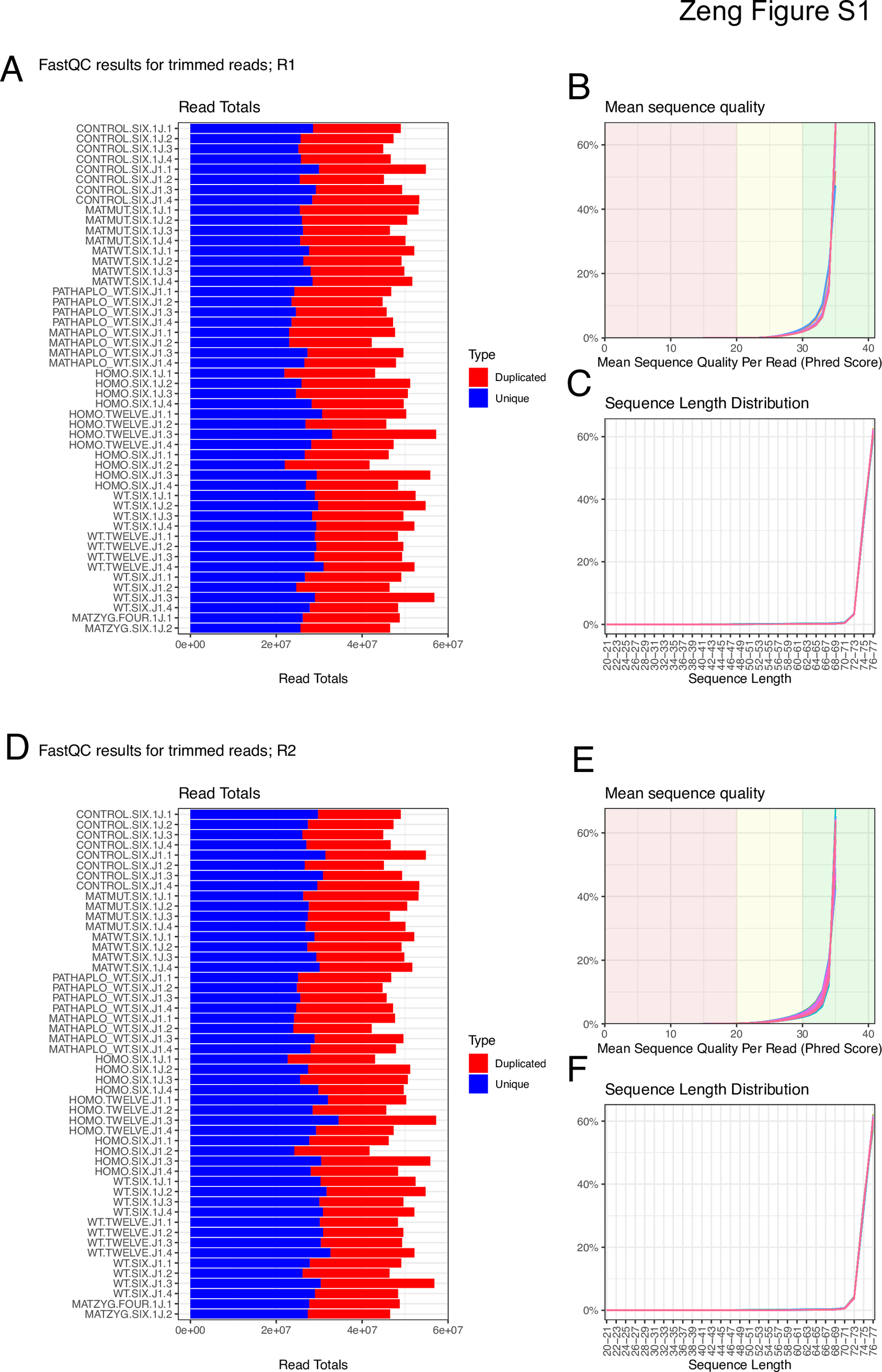

Supplement: S1 Fig — FastQC analysis is shown. (A) Total reads, unique and duplicated reads are plotted for each embryo sample in the R1 direction. (B) Mean sequence quality is plotted for each embryo sample in the R1 direction. (C) Sequence length distribution is plotted for each embryo sample in the R1 direction. (D) Total reads, unique and duplicated reads are plotted for each embryo sample in the R2 direction. (E) Mean sequence quality is plotted for each embryo sample in the R2 direction. (F) Sequence length distribution is plotted for each embryo sample in the R4 direction. (TIF) [file pgen.1009908.s001.tif]

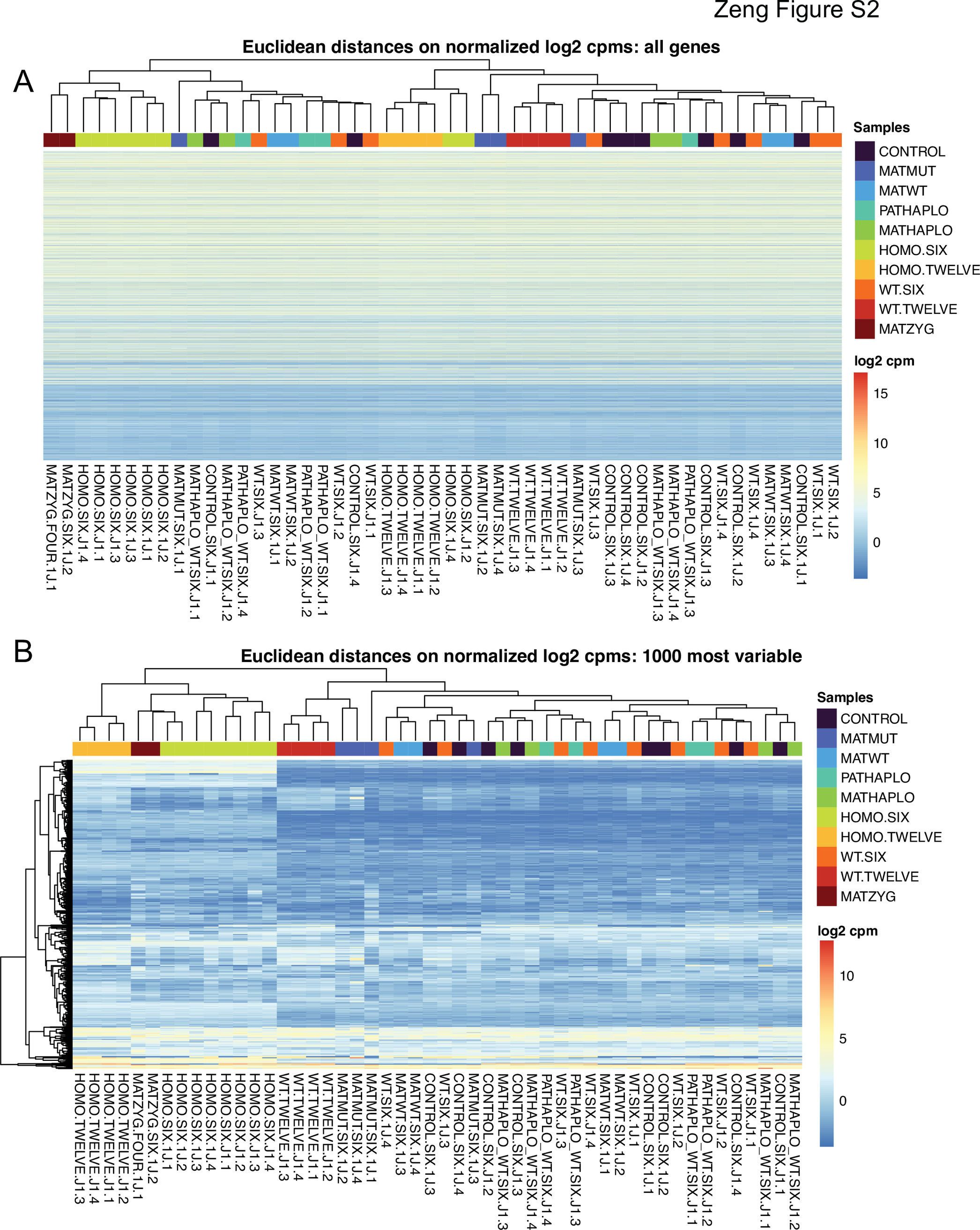

Supplement: S2 Fig — (A) Heatmap displays the results of an unsupervised cluster analysis that calculates Eucledian distance between samples based on all transcripts. Sample types are color-coded and normalized log2 cpm values are color-coded as shown to the right. Sample IDs are given at the bottom. Note the uniformity of the dataset. (A) Heatmap displays the results of an unsupervised cluster analysis that calculates Eucledian distance between samples based on the top 1000 variable transcripts. Note the split of the HOMO and MATZYG samples from the remaining samples. Also note the high variability of the HOMO6, MATZYG6, and MATMUT samples. (TIF) [file pgen.1009908.s002.tif]

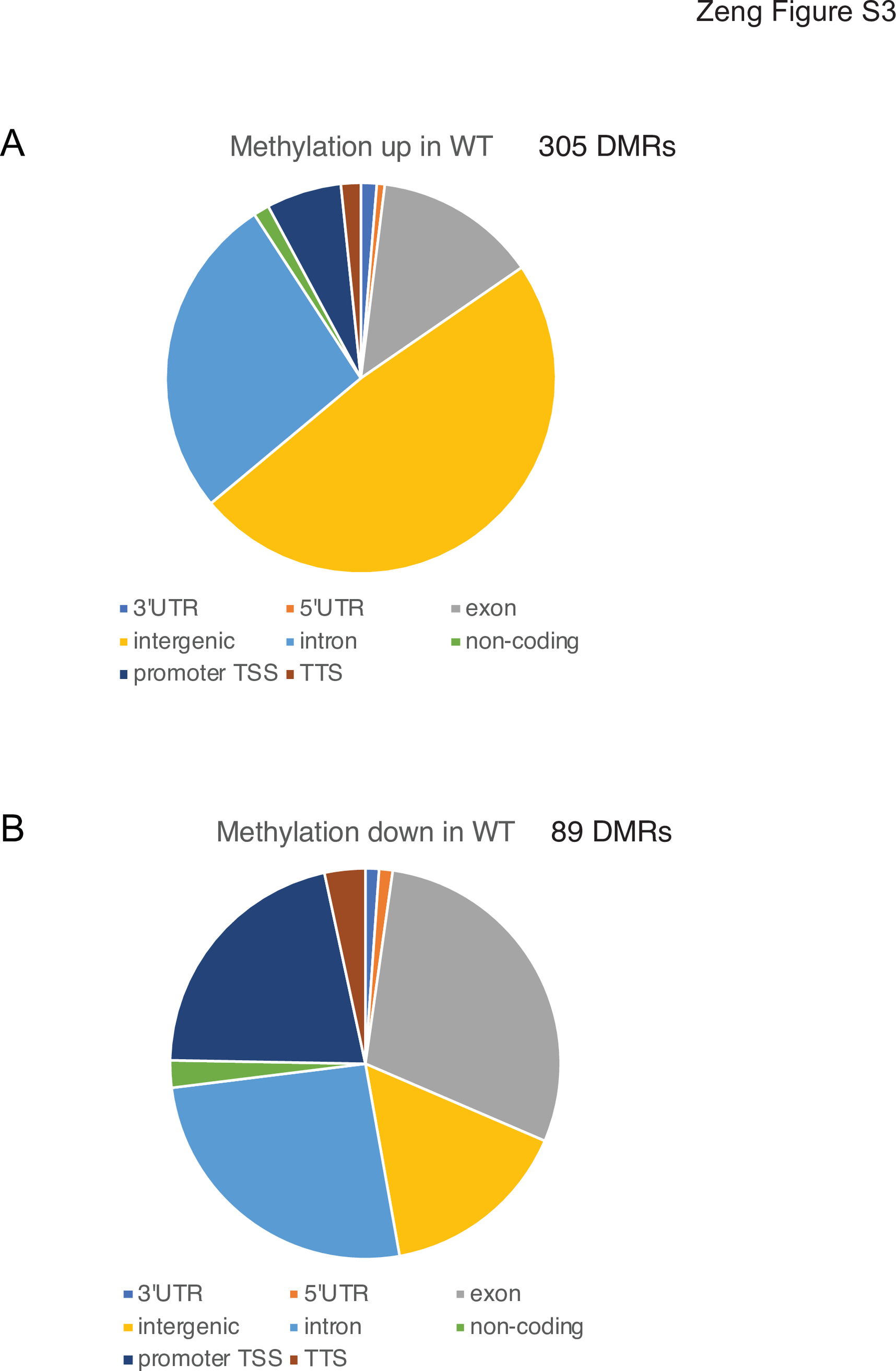

Supplement: S3 Fig — A WGBS experiment was performed comparing 9.5 dpc WT and HOMO embryos. DMRs were called and stratified into genomic elements as shown by colors. (A) DMRs that require EHMT2 for DNA hypermethylation. (B) DMRs that require EHMT2 for DNA hypomethylation. (TIF) [file pgen.1009908.s003.tif]

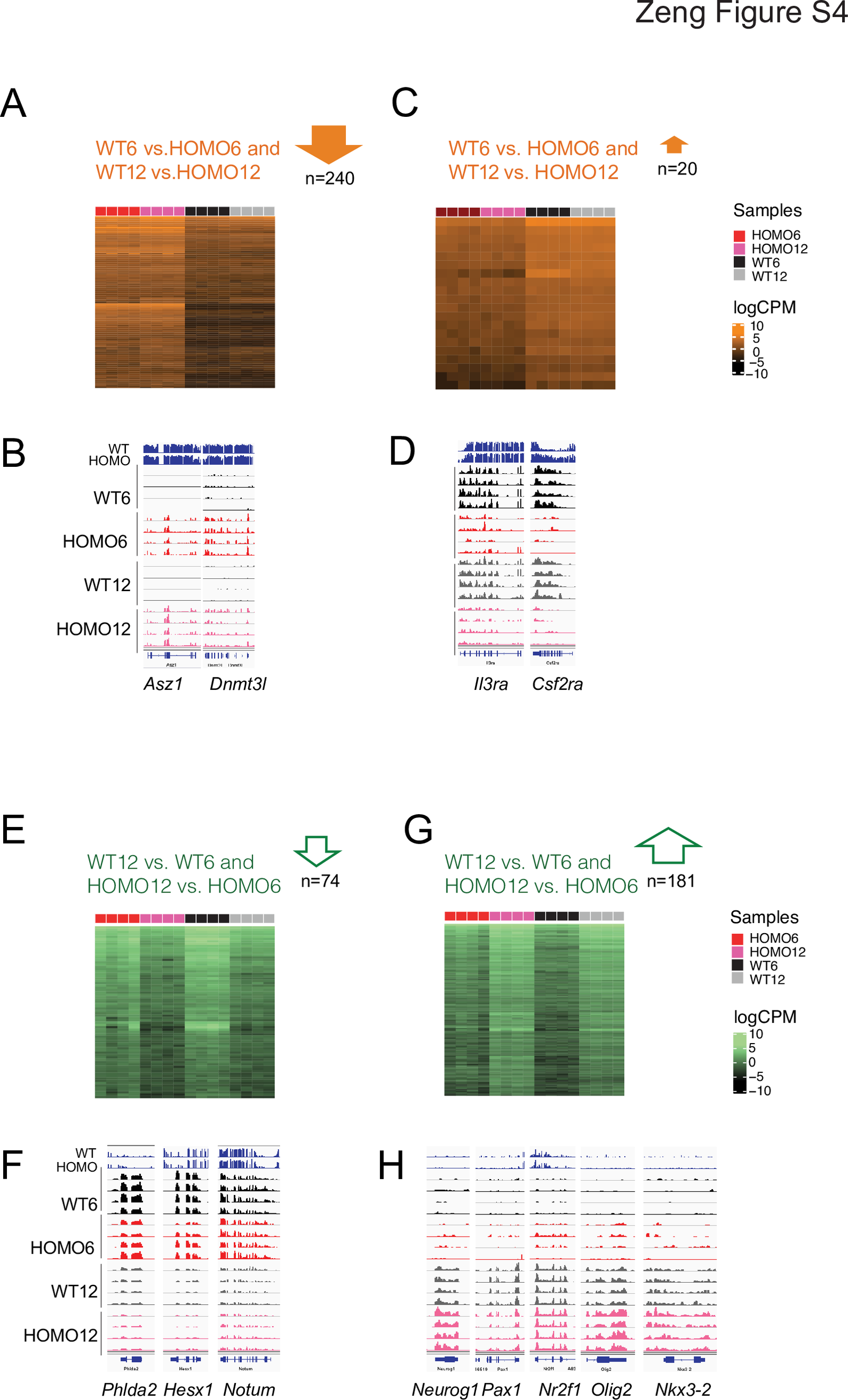

Supplement: S4 Fig — Heatmaps display the DE genes from the respective segments of the 4-way Venn diagrams depicted in Fig 3D and 3E. Arrows indicated the direction of changes with the number of DE genes in those sections. Below the heatmaps IGV browser examples are shown for the DEGs that drive the GO term in those specific Venn segments. Samples are labeled at the top with the color code shown to the right. Expression values (logCPM) are shown according to the scale to the right. (A-D) What it takes to be normal. Changes occur in response to EHMT2 at both the 6-somite and 12-somite stages. (E-H) What it takes to develop. Developmental DEGs downregulated (E) or upregulated (F) between the 6-and 12-somite stages regardless of EHMT2. Other details are as explained in Fig 3. (TIF) [file pgen.1009908.s004.tif]

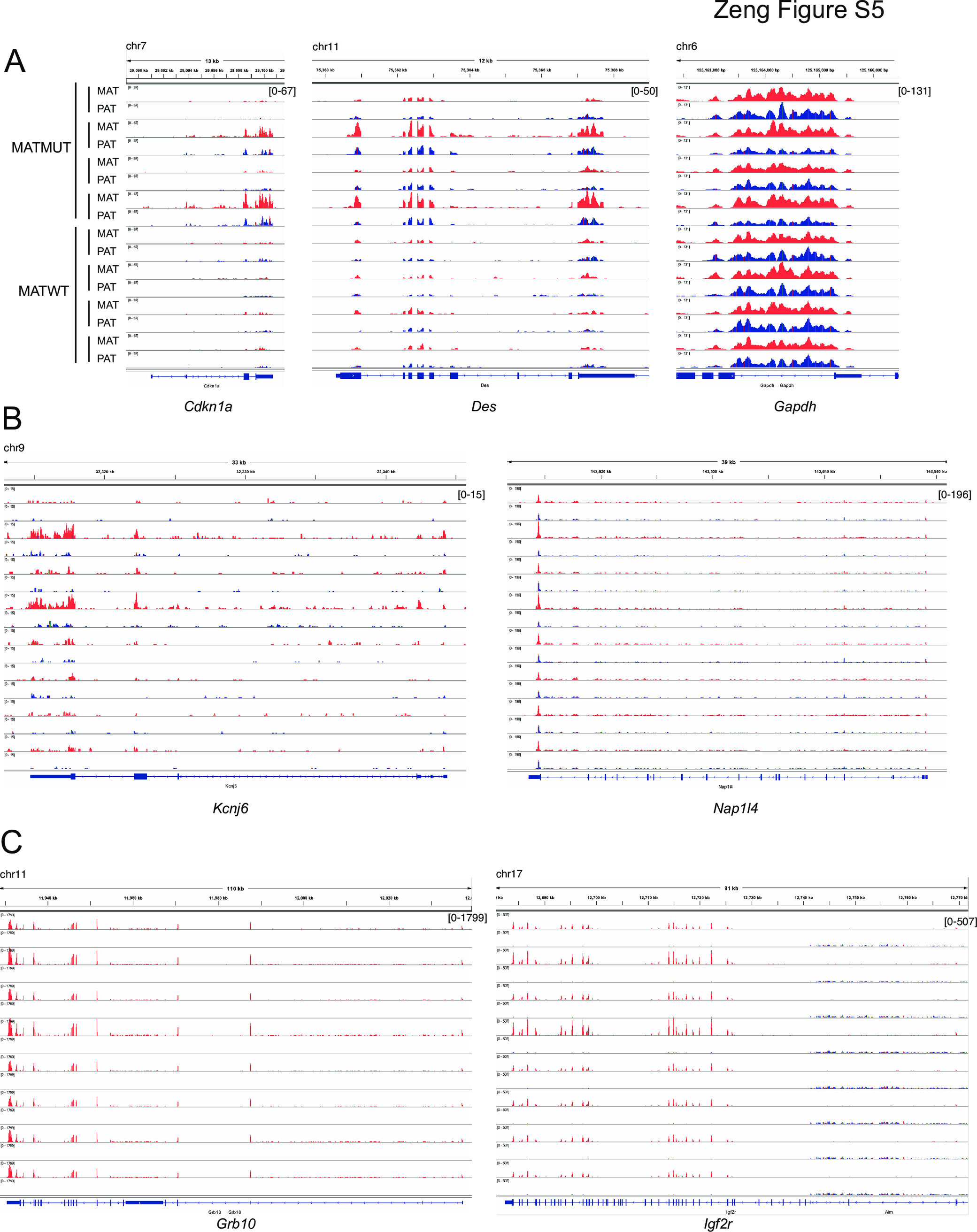

Supplement: S5 Fig — IGV browser images of the maternal (MAT) and paternal (PAT) alleles are shown of each representative transcript that exhibit high variability in MATMUT embryos in four replicate samples per each genotype. The transcription profile of the maternal (MAT) and paternal (PAT) alleles are shown of each transcript in four replicate samples per each genotype. (A), Both alleles are derepressed in MATMUT embryos, and the maternal allele is more derepressed than the paternal allele (B) The control gene Gapdh shows no variation in MATMUT embryos. (C) The maternal allele is derepressed in MATMUT embryos while the paternal allele is unchanged. (D) The maternal allele of imprinted genes is derepressed in MATMUT embryos while the paternal allele remains silent. (TIF) [file pgen.1009908.s005.tif]

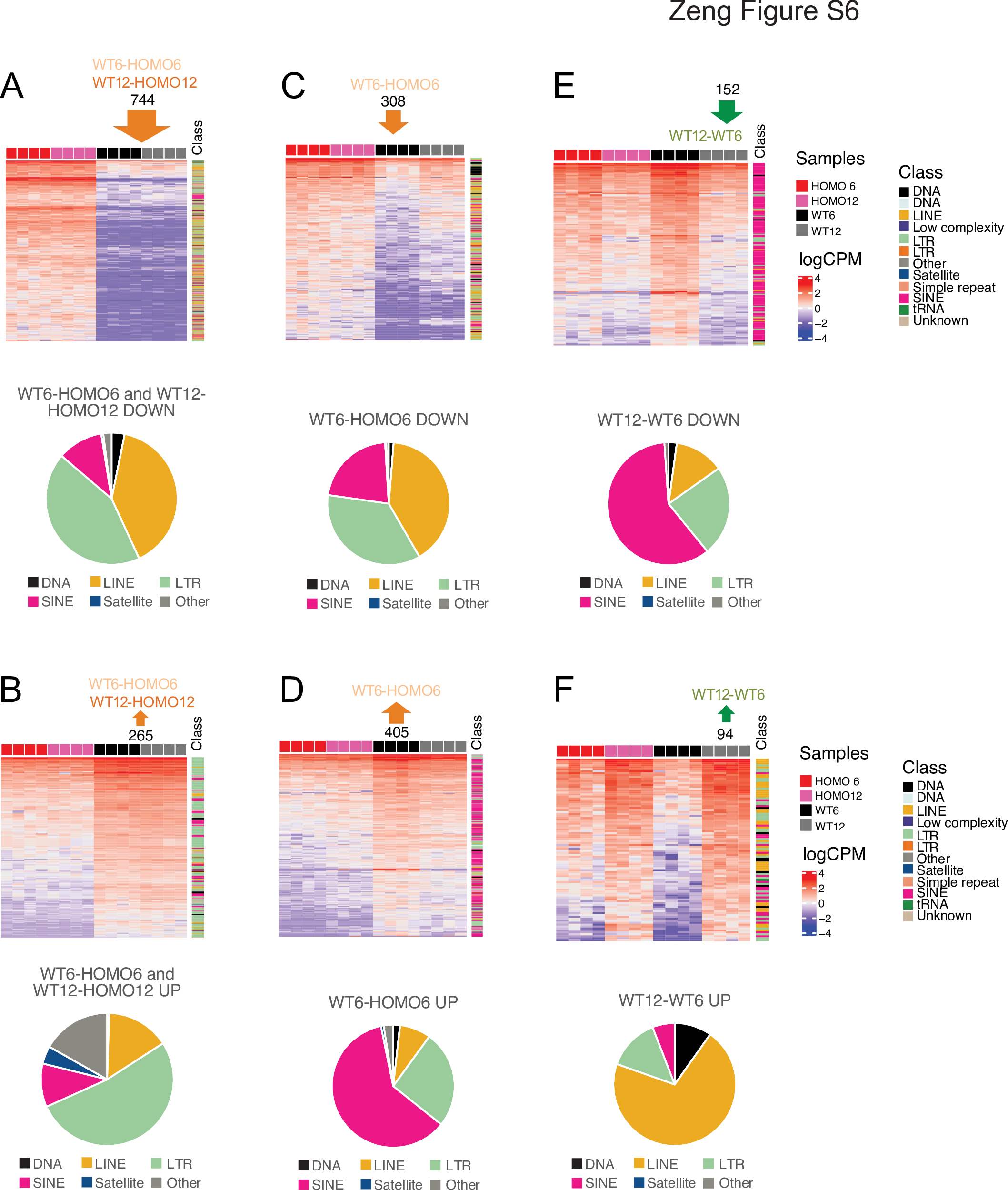

Supplement: S6 Fig — Uniquely mapped DE repeats were identified in the four-way comparison. (A-F) Heatmaps display the transcription level (logCPM) of those uniquely mapped repeats that are differentially expressed in the comparisons indicated at the top. Samples are shown by the color code to the right. The classification of each DE repeat is indicated in the pie charts underneath the respective heatmaps. (TIF) [file pgen.1009908.s006.tif]

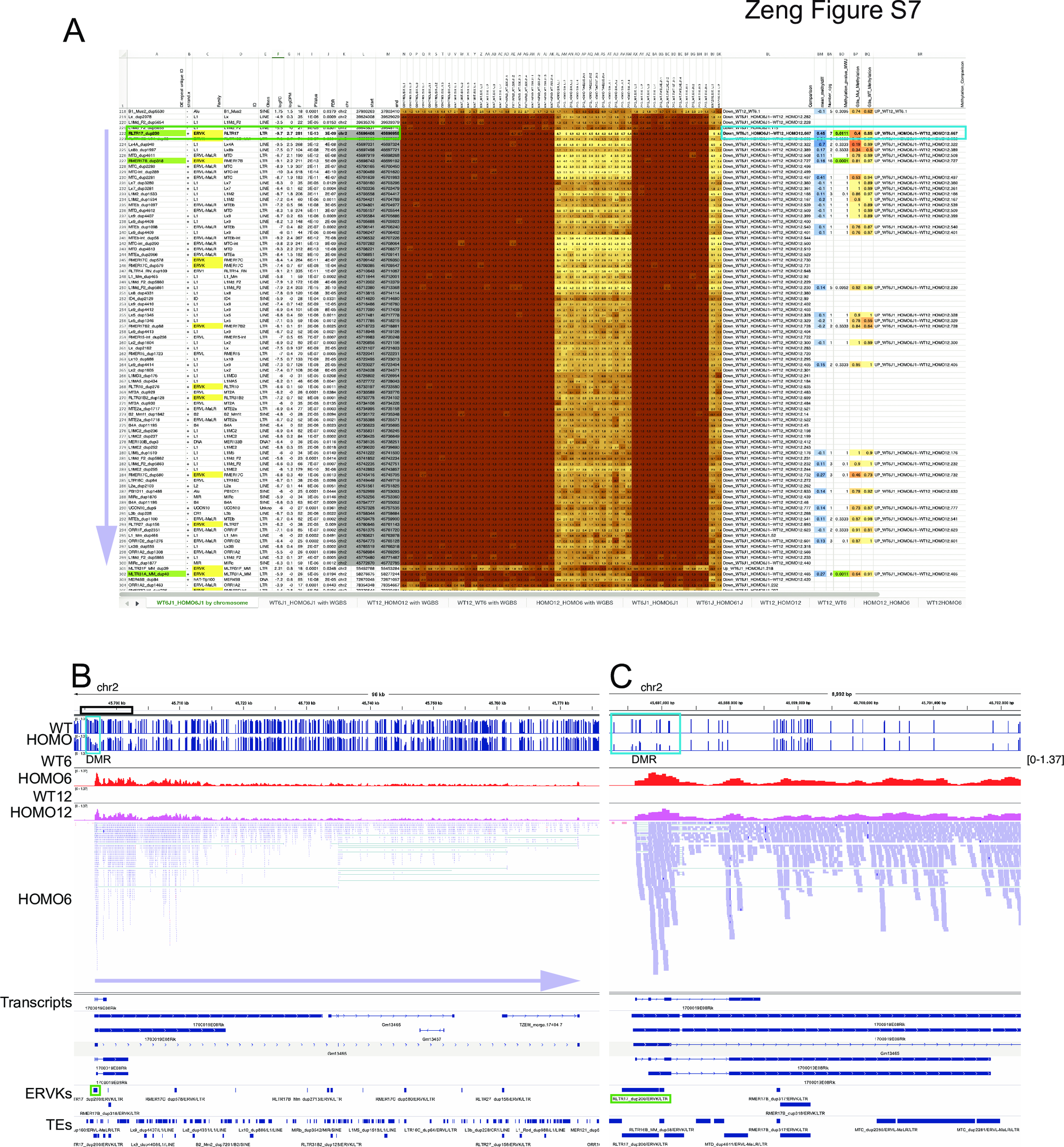

Supplement: S7 Fig — (A) Heatmap of differentially expressed TEs (as marked to the left) is depicted in the samples (as marked at the top) along a segment of chromosome 2. DNA methylation is displayed at TE-DMRs in WT and HOMO embryos (to the right). A long noncoding transcript is predicted (gray shading) by the synchronously misregulated ‘passenger’ repeats. It starts in an RLTR17 ERVK repeat (green rectangle), which is a DMR (turquoise rectangle), and it encompasses multiple ‘passenger’ repeats, which also appear to be DE repeats, irrespective of their initial directionality (strand). Blue vertical arrow indicates the long noncoding transcript that is defined by sequencing reads in the sense direction, as shown below. (B) Transcription initiates from an ERVK DMR in HOMO6 and HOMO12 embryos. One example is shown. IGV browser images of WGBS and RNAseq experiments are displayed in samples indicated to the left. The sequencing reads are displayed for the HOMO6 sample. Tracks of Refseq transcripts, putative transcripts, ERVKs, TEs, and DMRs are also included as marked to the left. Blue horizontal arrow indicates the long noncoding transcript that is defined by sequencing reads in the sense direction. It starts in an RLTR17 ERVK repeat (green rectangle), which is a DMR (turquoise rectangle), and it matches the putative transcript Gm13467. It encompasses multiple ‘passenger’ repeats, which also appear to be DE repeats, but which only display sequencing reads in the sense direction (blue) irrespective of their initial directionality. Part of the image is marked with black rectangle to be shown in more detail to the right. (C) Enlarged detail shows the initiation of the long noncoding transcript. (TIF) [file pgen.1009908.s007.tif]

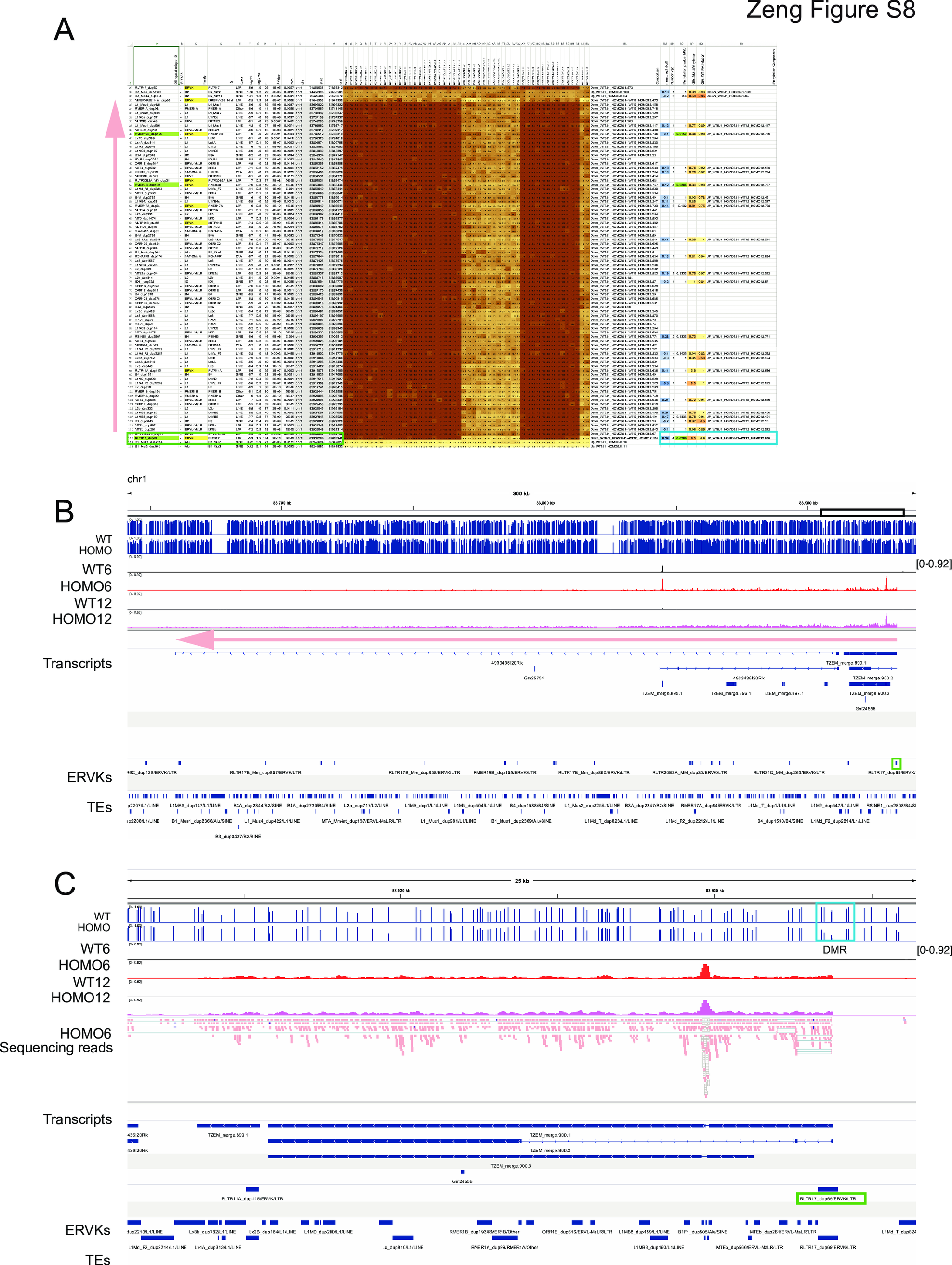

Supplement: S8 Fig — (A) Heatmap of differentially expressed TEs (as marked to the left) is depicted in the samples (as marked at the top) along a segment of chromosome 1. DNA methylation is displayed at TE-DMRs in WT and HOMO embryos (to the right). A long noncoding transcript is predicted (gray shading) by the synchronously misregulated ‘passenger’ repeats. It starts in an RLTR17 ERVK repeat (green rectangle), which is a DMR (turquoise rectangle), and it encompasses multiple ‘passenger’ repeats, which also appear to be DE repeats, irrespective of their initial directionality (strand). Pink vertical arrow indicates the long noncoding transcript that is defined by sequencing reads in the antisense direction, as shown below. (B) Transcription initiates from an ERVK DMR in HOMO6 and HOMO12 embryos. One example is shown. IGV browser images of WGBS and RNAseq experiments are displayed in samples indicated to the left. The sequencing reads are displayed for the HOMO6 sample. Tracks of Refseq transcripts, putative transcripts, ERVKs, TEs, and DMRs are also included as marked to the left. Blue horizontal arrow indicates the long noncoding transcript that is defined by sequencing reads in the sense direction. It starts in an RLTR17 ERVK repeat (green rectangle), which is a DMR (turquoise rectangle), and it matches the putative transcript 4933436I20Rik. It encompasses multiple ‘passenger’ repeats, which also appear to be DE repeats, but which only display sequencing reads in the antisense direction irrespective of their initial directionality. Part of the image is marked with black rectangle to be shown in more detail below. (C) Enlarged detail shows the initiation of the long noncoding transcript. (TIF) [file pgen.1009908.s008.tif]
